# Transcriptome-wide association study identifies new susceptibility genes and pathways for spondyloarthritis

**DOI:** 10.1186/s13018-023-04029-4

**Published:** 2023-09-04

**Authors:** Xiaochen Su, Anfa Chen, Menghao Teng, Wenchen Ji, Yingang Zhang

**Affiliations:** Department of Orthopaedics of the First Affiliated Hospital, Medical School, Xi’an Jiaotong University, Xi’an, 710061 Shaanxi China

**Keywords:** Spondyloarthritis, Transcriptome-wide association study, Differentially expressed gene, Pathway enrichment analysis, Gene ontology

## Abstract

**Background:**

Spondyloarthritis (SpA) is a group of multifactorial bone diseases influenced by genetic factors, the environment and lifestyle. However, current studies have found a limited number of SpA-related genes, and the genetic and pathogenic mechanisms of SpA are still unclear.

**Methods:**

A tissue-specific transcriptome-wide association study (TWAS) of SpA was performed using GWAS (including 3966 SpA patients and 448,298 controls) summary data and gene expression weights of whole blood and skeletal muscle. The SpA-associated genes identified by TWAS were further compared with the differentially expressed genes (DEGs) identified in the SpA gene expression profile acquired from the Gene Expression Omnibus database (GEO, GSE58667). Finally, functional enrichment and annotation analyses of the identified genes were performed.

**Results:**

The TWAS detected 499 suggestive genes associated with SpA in whole blood and skeletal muscle, such as *CTNNAL1* (*P*_SM_ = 3.04 × 10^−2^, *P*_WB_ = 9.58 × 10^−3^). The gene expression profile of SpA identified 20 candidate genes that overlapped in the TWAS data, such as *MCM4* (*P*_TWAS_ = 1.32 × 10^−2^, *P*_DEG_ = 2.75 × 10^−2^) and *KIAA1109* (*P*_TWAS_ = 3.71 × 10^−2^, *P*_DEG_ = 4.67 × 10^−2^). Enrichment analysis of the genes identified by TWAS identified 93 significant GO terms and 33 KEGG pathways, such as mitochondrion organization (GO: 0007005) and axon guidance (hsa04360).

**Conclusion:**

We identified multiple candidate genes that were genetically related to SpA. Our study may provide novel clues regarding the genetic mechanism, diagnosis, and treatment of SpA.

**Supplementary Information:**

The online version contains supplementary material available at 10.1186/s13018-023-04029-4.

## Highlights

1. This is the first study to identify susceptibility genes associated with spondyloarthritis (SpA), revealing 499 suggestive genes by TWAS analysis.
2. The TWAS results were further verified by comparing the gene expression profiles.
3. This study provides novel clues for understanding the genetic mechanism of SpA, especially focusing on aberrant gene expression in the pathogenesis of SpA.

**Supplementary Information:**

The online version contains supplementary material available at 10.1186/s13018-023-04029-4.

## Introduction

Spondyloarthritis (SpA) is a group of related but phenotypically distinct disorders, including psoriatic arthritis, arthritis related to inflammatory bowel disease, reactive arthritis, juvenile idiopathic arthritis, and ankylosing spondylitis [1, 2]. The symptoms of SpA include inflammation of the axial skeleton, asymmetric peripheral arthritis of the lower limbs, enthesitis, and extra-articular manifestations, and all subtypes of SpA share a common genetic background [3, 4]. The prevalence of SpA shows considerable differences among ethnic groups and populations that can vary from 0.01% (Japan) to 2.5% (Alaska), and the annual estimated incidence of SpA is 62.5/100,000. The worldwide prevalence of SpA has seriously affected the function of the musculoskeletal system and reduced the quality of life [4, 5]. Recently, an increasing number of studies have focused on the genetic mechanisms of SpA [6, 7]. Through familial aggregation, previous studies have estimated that genetic risk factors contribute to 80–90% of the susceptibility to SpA such as ankylosing spondylitis [7].

Genome-wide association studies (GWASs) have been considered to be one of the primary approaches for determining genetic links to diseases [8, 9]. By a GWAS approach, Díaz-Peña et al. [10] revealed the potential involvement of mechanisms and pathways that were previously unsuspected in SpA, particularly those regarding aminopeptidases or the *IL23/IL17* pathways. However, the GWAS is only recommended for evaluating the risk of disease. Because most GWAS-identified single nucleotide polymorphisms (SNPs) are located in the noncoding regions of the genome, so the interpretation of those variants at the gene expression level is limited [11]. Expression quantitative trait loci (eQTL) analysis is a way to identify genes related to variations in gene expression [12]. Therefore, integrating GWAS and eQTL analysis may help more effectively to identify candidate genes associated with disease. In the previous study, researcher integrated publicly available GWAS summary data and eQTL reference data sets to evaluate gene-trait relationships, an approach referred to as the transcriptome-wide association study (TWAS) [13]. Different from GWAS, TWAS can drastically reduce the comparisons in statistical analysis and enhance the ability to detect candidate genes of target diseases [14].

In recent years, TWAS has been widely used to identify genetic loci associated with target diseases. For example, Liao et al. [15] identified 9 transcriptome-wide significant hits associated with attention deficit/hyperactivity disorder, of which 6 genes were not implicated in the original GWAS. In addition, Mancuso N et al. identified 217 genes at 84 independent 1 Mb regions associated with prostate cancer risk through TWAS analysis, which could provide novel risk loci and prioritize putative causal genes at known risk loci associated with prostate cancer [16]. However, only few GWAS and no TWAS analyses focus on SpA. Therefore, we identify candidate genes to reveal the genetic and pathogenic mechanisms of SpA.

## Materials and methods

### SpA GWAS summary data set

The large-scale SpA GWAS summary data was obtained from a published study [17]. In short, the data set contained 3966 diagnosed SpA patients and 448,298 controls of European ethnicity from the UK Biobank (https://biobank.ndph.ox.ac.uk/showcase/field.cgi?id=20002. Accessed 19 March 2021) (Additional file 1) [17]. The UK Biobank participants were genotyped using the Affymetrix UK Bileve AXIOM or UK Biobank AXIOM array and imputed against approximately 90 million genetic variations from the Haplotype Reference Consortium, 1000 Genomes and the UK 10 K Project [17]. After filtering, the data set contained 9,113,133 imputed variants. The IMPUTE4 program was used to perform the imputation (http://jmarchini.org/software/). Detailed information on the subjects, genotyping, imputation, and quality control can be found in a published study (Additional file 2) [17]. In addition, we performed additional quality controls on the above GWAS data: (1) removal of single nucleic acid polymorphisms (SNPs) without rsID; (2) uniform alignment with the hg19 human reference genome.

### TWAS of SpA

The TWAS of SpA was carried out by functional summary-based imputation software (FUSION http://gusevlab.org/projects/fusion/). FUSION is a new approach to identify genes whose expression is significantly associated with complex traits in individuals without directly measuring the expression level by integrating GWAS summary data and precalculated gene expression weights of different tissues [18]. The whole blood and skeletal muscle tissues were also used in previous biological studies of SpA [19]. In this study, we used the pre-calculated gene expression weights of whole blood and skeletal muscle by the FUSION prediction models. Then, the calculated gene expression weights were combined with GWAS statistics to impute the association statistics between the gene expression level and SpA. FUSION software and the gene expression weight panels for skeletal muscle and whole blood were downloaded from the FUSION website (http://gusevlab.org/projects/fusion/). For TWAS results, a significant association threshold of *P* < 5.38 × 10^−6^ (0.05/9300) after strict Bonferroni correction was adopted. *P* values between *P* < 5.38 × 10^−6^ and 0.05 were considered suggestive of significance [20, 21].

### Gene expression profile of SpA

The gene expression profile of SpA was acquired from the Gene Expression Omnibus (GEO) database (https://www.ncbi.nlm.nih.gov/GEO/, accession number: GSE58667). Briefly, DNA microarray-based gene expression levels were examined in peripheral blood from 11 patients with juvenile SpA (jSpA) patients and 4 healthy controls from Croatia, along with a bioinformatics analysis of the retrieved data, and the carefully selected differentially expressed genes (DEGs) of all participants in the study were analyzed by qRT-PCR [22]. The GEO2R tool was used to identify the DEGs. GEO2R presents a simple interface that allows users to perform sophisticated R-based analysis of GEO data to identify and visualize differential gene expression [23]. Genes were identified as differentially expressed when the following two conditions were met: *P* value < 0.05 by the moderated t statistic and |log2FC|> 1 [22].

### Gene annotation analysis and functional enrichment analysis

In this study, we used the Metascape (https://metascape.org/gp/index.html) tool to perform Gene Ontology (GO) and pathway enrichment analyses of candidate genes identified by the TWAS and gene expression profiles [24]. The significant GO terms and Kyoto Encyclopedia of Genes and Genomes (KEGG) pathways were screened out by comparing the results of TWAS analysis and the gene expression profiles. Then, the functions of significant genes shared by TWAS analysis and gene expression profiles were annotated, prioritized, visualized, and interpreted by the Functional Mapping and Annotation of GWAS (FUMA https://fuma.ctglab.nl/) tool [25].

## Results

### TWAS results of SpA

**Fig. 1:**
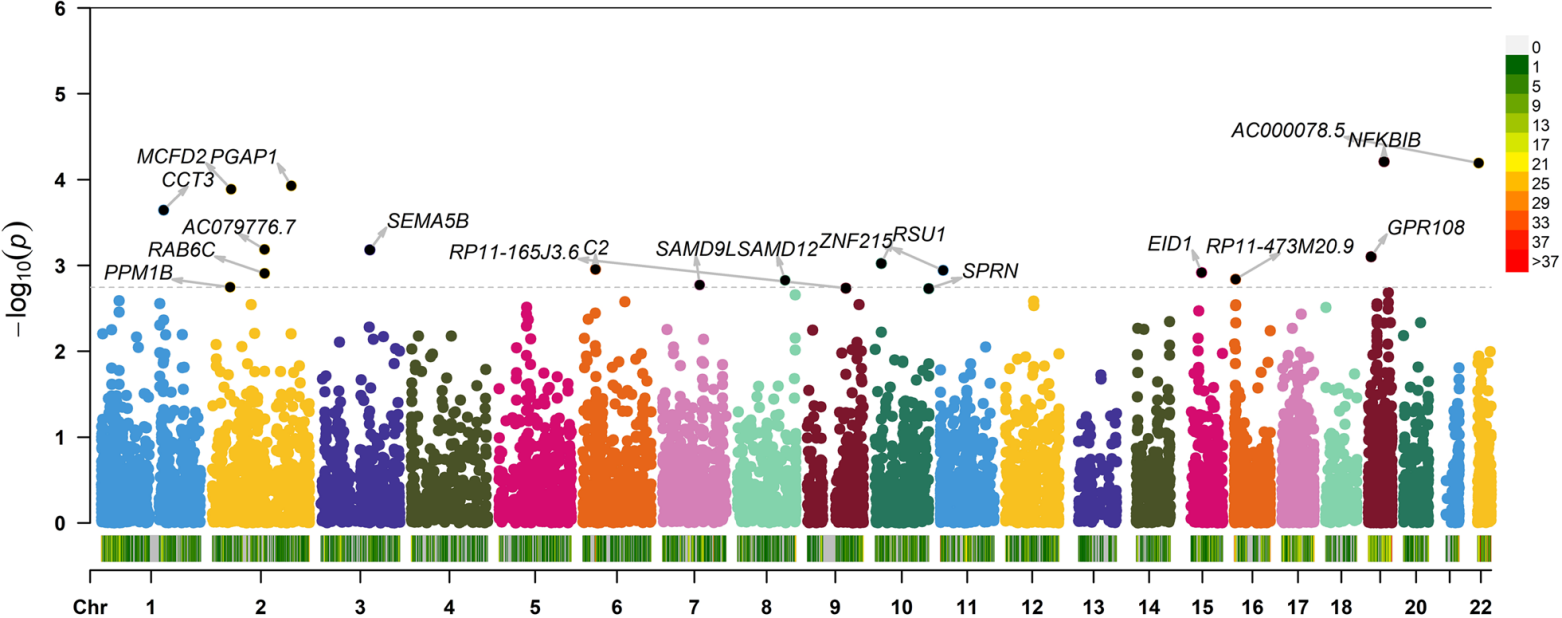
Manhattan plot showing TWAS-identified genes. Note: Manhattan plot showing TWAS-identified genes and significantly expressed genes associated with SpA (annotated points). Each point represents a single gene, and the physical position (chromosome localization) is plotted on the *x*-axis, while the − log10 (*P* value) of the association between gene and SpA is plotted on the *y*-axis. TWAS: Transcriptome-wide association study; SpA: spondyloarthritis

TWAS analysis identified 390 suggestive genes in skeletal muscle and 137 suggestive genes in whole blood, with *P* values between 5.38 × 10^–6^ and 0.05 (Fig. 1, Additional file 3: Table S1). In addition, 28 genes were identified in both skeletal muscle and whole blood, including cadherin-associated protein, alpha-like 1 (*CTNNAL1*) (*P*_SM_ = 3.04 × 10^−2^, *P*_WB_ = 9.60 × 10^−3^), *AC000078.5* (*P*_SM_ = 1.17 × 10^−2^, *P*_WB_ = 1.00 × 10^−4^ 0.0001), *RP11-165J3.6* (*P*_SM_ = 1.86 × 10^−2^, *P*_WB_ = 1.84 × 10^−3^) and *ZNF100* (*P*_SM_ = 3.00 × 10^−3^, *P*_WB_ = 2.80 × 10^−3^). Table 1 presents detailed information on the top significant genes identified by TWAS, including the TWAS P value, TWAS Z score, and number of SNPs in the locus (NSNP).

**Table 1:** The common genes identified by TWAS analysis

Gene	CHR	Skeletal muscle			Whole blood		
		NSNP	TWAS.Z	TWAS.*P*_SM_	NSNP	TWAS.Z	TWAS.*P*_WB_
*AC000078.5*	22	551	− 2.5198	6.42 × 10^−2^	514	− 3.9967	6.42 × 10^−5^
*RP11-165J3.6*	9	493	− 2.3532	1.86 × 10^−2^	473	− 3.1153	1.86 × 10^−3^
*ZNF100*	19	298	2.9700	2.98 × 10^−3^	295	2.9885	2.80 × 10^−3^
*ZNF493*	19	286	2.8573	4.27 × 10^−3^	283	2.8464	4.42 × 10^−3^
*ZNF429*	19	299	2.8573	4.27 × 10^−3^	304	2.7375	6.19 × 10^−3^
*STK17B*	2	371	− 2.1650	3.04 × 10^−2^	376	− 2.7341	6.26 × 10^−3^
*ZC3H3*	8	360	3.0619	2.20 × 10^−3^	353	2.6965	7.01 × 10^−3^
*ZNF738*	19	285	2.2961	2.17 × 10^−2^	276	2.6729	7.52 × 10^−3^
*CTNNAL1*	9	622	2.1645	3.04 × 10^−2^	633	2.5905	9.58 × 10^−3^
*GGTA1P*	9	442	2.6587	7.84 × 10^−3^	445	− 2.5057	1.22 × 10^−2^
*RP11-254F7.2*	2	579	− 2.4359	1.49 × 10^−2^	555	− 2.5043	1.23 × 10^−2^
*ARFGAP3*	22	531	2.1865	2.88 × 10^−2^	532	− 2.4671	1.36 × 10^−2^
*KLHL12*	1	490	− 2.7270	6.39 × 10^−3^	471	2.4202	1.55 × 10^−2^
*SRR*	17	409	− 2.2354	2.54 × 10^−2^	406	− 2.4130	1.58 × 10^−2^
*RP11-611E13.2*	12	575	2.4076	1.61 × 10^−2^	763	2.4076	1.61 × 10^−2^
*NT5C3B*	17	409	− 2.3002	2.14 × 10^−2^	399	− 2.3818	1.72 × 10^−2^
*MBLAC1*	7	281	− 2.0665	3.88 × 10^−2^	265	− 2.3050	2.12 × 10^−2^
*BNIP1*	5	579	− 2.1862	2.88 × 10^−2^	566	− 2.2589	2.39 × 10^−2^
*LRRC61*	7	348	2.0121	4.42 × 10^−2^	344	2.2517	2.43 × 10^−2^
*CCDC125*	5	235	− 2.2450	2.48 × 10^−2^	250	− 2.2383	2.52 × 10^−2^
*CHD1 L*	1	407	− 2.5622	1.04 × 10^−2^	427	2.2280	2.59 × 10^−2^
*RP11-218M22.1*	12	457	− 2.0853	3.70 × 10^−2^	452	− 2.2221	2.63 × 10^−2^
*HLA-DQA1*	6	214	− 2.0896	3.67 × 10^−2^	210	− 2.1745	2.97 × 10^−2^
*ZNF205*	16	385	− 2.0686	3.86 × 10^−2^	363	− 2.1201	3.40 × 10^−2^
*POLR2J3*	7	228	− 2.2285	2.59 × 10^−2^	228	− 2.0773	3.78 × 10^−2^
*RAPGEFL1*	17	295	− 2.1276	3.34 × 10^−2^	290	− 2.0455	4.08 × 10^−2^
*NUDCD3*	7	352	− 2.4406	1.47 × 10^−2^	371	− 2.0091	4.45 × 10^−2^
*AC091729.9*	7	400	2.0595	3.95 × 10^−2^	378	2.0062	4.48 × 10^−2^

The SpA GWAS summary data set and the pre-calculated reference weights of gene expression profiles in whole blood and skeletal muscle were used for TWAS analysis of SpA. Each TWAS.P value was calculated by TWAS analysis (http://gusevlab.org/projects/fusion/)

TWAS: Transcriptome-wide association study; GWAS: genome-wide association study; SpA: spondyloarthritis; TWAS.*P*_SM_: TWAS *P*_Skeletal-muscle_ value; TWAS.*P*_WB_: TWAS *P*_Whole-Blood_ value; TWAS.Z: TWAS Z score; NSNP: number of SNPs in the locus; FUSION, functional summary-based imputation

### Validating TWAS results by gene expression profiles of SpA

**Fig. 2:**
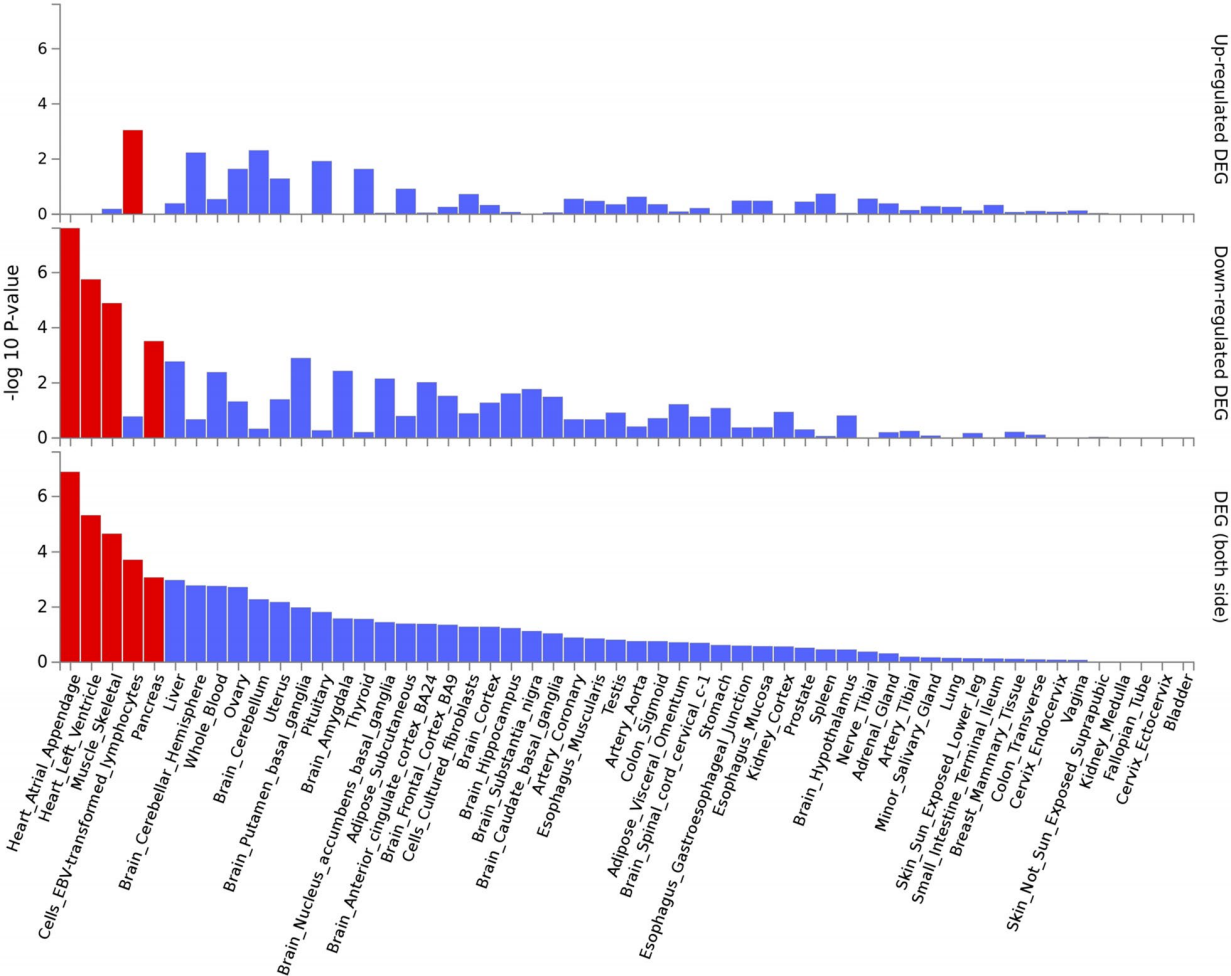
The expression of common spondyloarthritis (SpA) genes at different tissue sites. Note: Image of the expression of common genes identified by both TWAS analysis and gene expression profiling. The bars in red show significant differential expression. TWAS, Transcriptome-wide association study; SpA, spondyloarthritis

**Fig. 3:**
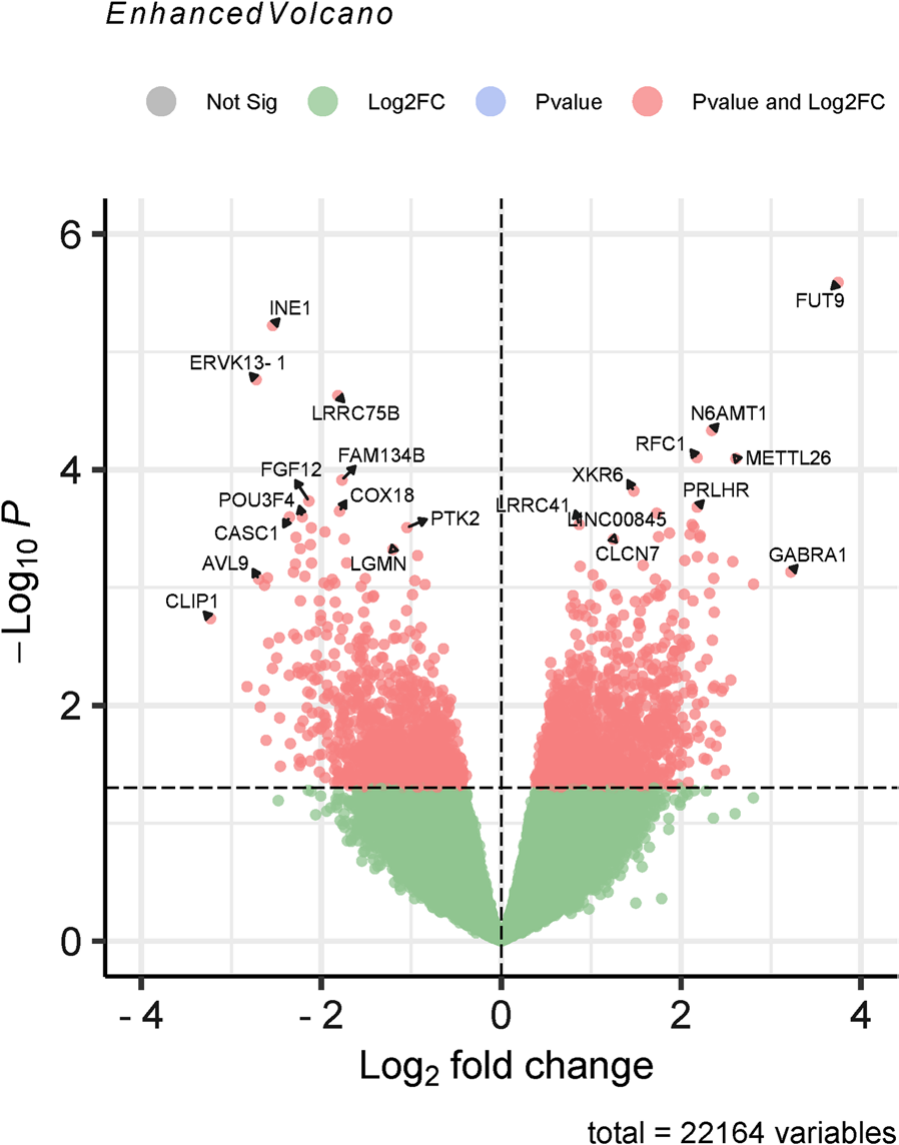
Volcano plot of gene expression profiles for spondyloarthritis (SpA). Note: The results of gene expression profiling were output in a volcano map. Genes marked in red were differentially expressed according to the following two conditions: *P* value of < 0.05 by the moderated t statistic and |logFC|> 1. DEG: Differentially expressed gene; SpA: spondyloarthritis

By comparing the genes identified by TWAS analysis and the gene expression profile of SpA, we screened 20 DEGs, such as *MCM4* (*P*_TWAS_ = 1.32 × 10^−2^, *P*_DEG_ = 2.75 × 10^−2^), *KIAA110*9 (*P*_TWAS_ = 3.71 × 10^−2^, *P*_DEG_ = 4.67 × 10^−2^) and *SFMBT2* (*P*_TWAS_ = 2.94 × 10^−2^, *P*_DEG_ = 2.45 × 10^−2^) (Table 2). Using FUMA software, we found that 20 candidate genes were differentially expressed in the musculoskeletal system. In addition, the expression of common genes was significantly downregulated in muscle-skeletal tissue (− log 10 *P* value > 4, Fig. 2). The distribution of gene expression values in the gene expression profiles was visualized in the corresponding volcano plot (Fig. 3).

**Table 2:** The common genes identified by both TWAS analysis and gene expression profiles of SpA

Tissue	Gene	CHR	*P*_TWAS_	TWAS.Z	*P*_DEG_	Regulation
Skeletal muscle	*MCM4*	8	3.37 × 10^–2^	− 2.1232	1.61 × 10^–2^	Up
	*ATRNL1*	10	4.34 × 10^–2^	2.0195	2.56 × 10^–2^	Down
	*CWF19L2*	11	4.06 × 10^–2^	2.0481	4.69 × 10^–2^	Down
	*CYB5A*	18	1.83 × 10^–2^	2.3589	3.82 × 10^–2^	Down
	*ZNF738*	19	2.17 × 10^–2^	2.2961	3.07 × 10^–2^	Down
	*KIAA1109*	4	3.71 × 10^–2^	2.0843	4.67 × 10^–2^	Down
Whole blood	*SFMBT2*	10	2.94 × 10^–2^	− 2.1775	2.45 × 10^–2^	Up
	*FRA10AC1*	10	4.00 × 10^–2^	2.0535	1.49 × 10^–2^	Down
	*SAMD10*	20	2.24 × 10^–2^	− 2.2829	2.20 × 10^–2^	Up
	*SMG5*	1	4.30 × 10^–3^	− 2.8549	5.00 × 10^–3^	Up

Each *P*_TWAS_ value was calculated by TWAS Analysis. Each *P*_DEG_ value was the DEG derived from published studies

TWAS: Transcriptome-wide association study; DEG: differentially expressed gene; *P*_TWAS_: *P*_Transcriptome-Wide Association Study_ value; *P*_DEG_: *P*_Differentially Expressed Gene_ value; NSNP: number of SNPs in the locus

### Gene annotation analysis and functional enrichment

**Fig. 4:**
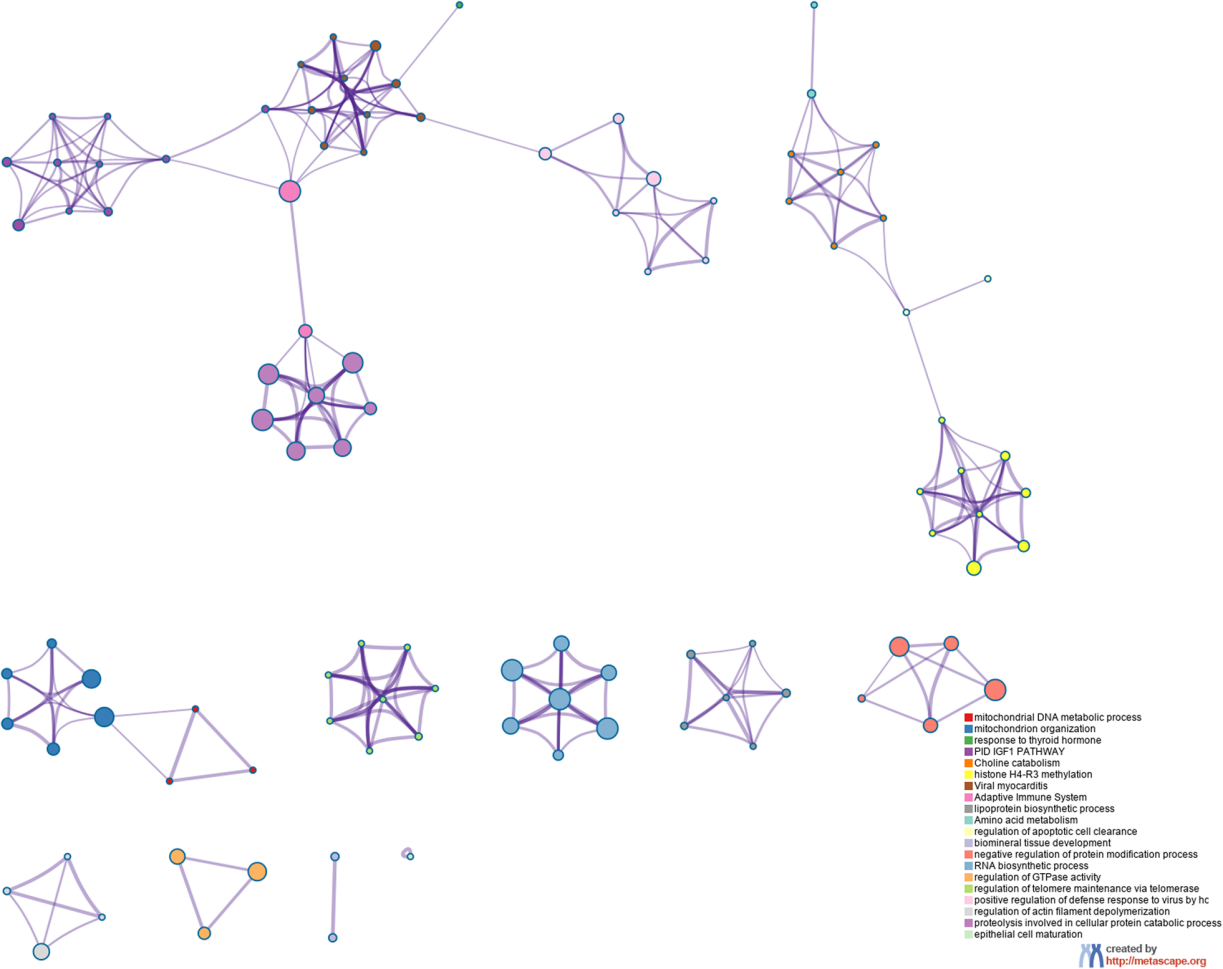
The network layout of representative Gene ontology (GO) terms. Note: The network layout of representative GO terms under hierarchical clustering. In the network, each circle node represents a term, where node size is proportional to the number of input genes associated with that term, and node color represents cluster identity (i.e., nodes of the same color belong to the same cluster). Terms with a similarity score > 0.3 are linked by an edge (the thickness of the edge represents the similarity score). GO: Gene ontology

**Fig. 5:**
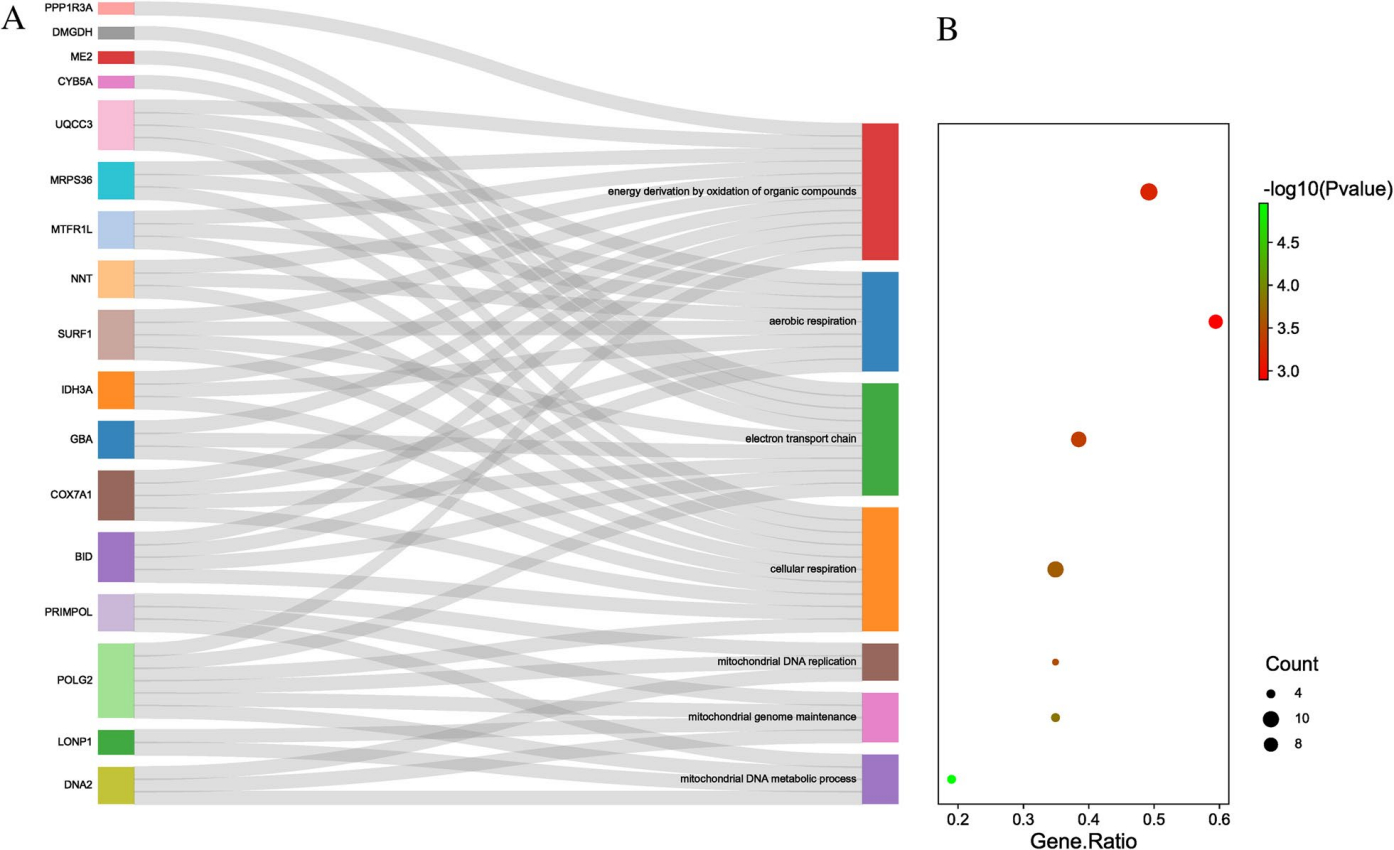
Top representative and overrepresented Gene ontology (GO) terms and related genes. **A** Note: The GO Chord plot of top overrepresented GO terms belonging to the Biological Process subontology for spondyloarthritis (SpA). The genes are linked to their assigned terms via colored ribbons. Genes are ordered according to the observed log FC, which is displayed in descending and ascending intensity of red and blue squares displayed next to the selected genes. GO: Gene ontology; SpA: spondyloarthritis; log FC: log fold change. **B** Note: Sankey plot showing the relationships between the genes and overrepresented GO terms. The dot plot shows the ratio between the genes found to be involved in GO terms and the total number of genes included in each GO term (FDR *P* ≤ 0.05). GO: Gene ontology

In this study, pathway and process enrichment analyses were carried out based on the following ontology resources: GO biological processes, KEGG pathways, GO molecular functions, Reactome gene sets, canonical pathways, and CORUM. The 528 genes identified by TWAS in the two tissues were successfully submitted to Metascape to perform gene enrichment analysis. The Metascape tool identified 93 GO terms that were enriched in SpA, such as mitochondrion organization (GO:0007005), generation of precursor metabolites and energy (GO:0006091), and histone H4-R3 methylation (GO:0043985). We also identified 33 KEGG pathways in SpA, such as axon guidance (hsa04360) and purine metabolism (hsa00230) (Additional file 4: Table S2). The significant terms were then hierarchically clustered, a subset of representative terms was selected, and the results were converted into a network layout (Fig. 4). Combined with Fig. 4 and Additional file 4: Table S2, the results indicated that the most significant biological pathways were antigen processing and presentation, the adaptive immune system, and mitochondrion organization. The GO Chord, Sankey and dot plots illustrating the top representative and overrepresented GO terms belonging to SpA are shown in Fig. 5A and B.

## Discussion

SpA is a group of complex trait diseases, and genetic susceptibility accounts for a large part of its pathogenesis. Previous studies have identified a large number of genetic loci associated with SpA, but their ability to interpret the relationship between the significant genes and SpA was limited [10]. To learn more about the potential genetic mechanisms of SpA, we conducted a TWAS analysis based on the large-scale GWAS summary data set acquired from a European cohort including 3966 SpA patients and 448,298 controls. Our results revealed a total of 499 potential disease-associated genes, including 390 suggestive genes identified in skeletal muscle and 137 suggestive genes identified in whole blood. This is the first study to identify genes associated with SpA by TWAS analysis.

The TWAS identified several genes associated with SpA, such as *SFMBT2*, *MCM4*, *KIAA1109*, and *CTNNAL1*. The *SFMBT2* protein is a member of the polycomb group (PcG) of proteins. Hussain et al. [26] found that *SFMBT2* interference altered the expression of key metabolic genes in chondrocytes; *SOX9* and *COL2A1* were decreased, whereas *MMP13* and *ADAMTS4* were significantly increased. Some studies have shown that upregulation or downregulation of these genes, which are altered by the *SFMBT2* gene, can lead to cartilage degeneration and further cause SpA [27, 28]. The *ATRNL1* gene, which was identified in this study, can regulate the expression of *SOX9* and is significantly highly expressed in the cartilage tissues of patients with osteoarthritis [29]. In summary, *SFMBT2* and *ATRNL1* may be associated with the genetic mechanisms of SpA pathogenesis. *SFMBT2* and *ATRNL1* may be candidate genes related to SpA, and the genetic mechanisms linking these genes and SpA need further research.

Another important candidate gene identified in this study was *MCM4*. The *MCM4* protein is a DNA replication licensing factor that is essential for DNA replication initiation and elongation in eukaryotic cells. In other words, the *MCM4* gene acts as an essential regulator of the cell cycle. *MCM4* can cause many diseases by regulating the cell cycle and inducing apoptosis [30, 31]. In addition, previous studies have shown that the pathogenesis of SpA includes thinning of the cartilage and cartilage degeneration, which involves chondrocyte apoptosis and proteoglycan loss [31].

In addition, *KIAA1109* was identified in both the TWAS and the gene expression profile of SpA. A study showed that a 480 kb block on chromosome 4q27 encompassing the *KIAA1109/Tenr/IL-2/IL-21* gene cluster is associated with rheumatoid arthritis [32]. Zhernakova et al. [33] found that the *KIAA1109/Tenr/IL-2/IL-21* gene cluster is involved in susceptibility to multiple autoimmune diseases, suggesting that this locus is a general risk factor for diseases such as rheumatoid arthritis and celiac disease. SpA is a subset of seronegative rheumatic-related autoimmune diseases, and Bowes et al. found significant evidence associating SpA susceptibility with the *IL-2* and *IL-21* genes [34, 35]. To the best of our knowledge, no researchers studied whether *KIAA1109* has a direct effect on SpA. Therefore, this is the first study exploring the genetic correlation between *KIAA1109* and SpA.

*CTNNAL1* is ubiquitously expressed in many tissues, including skeletal muscle, the pancreas, and the heart [36]. By comparing psoriasis patients who did not have psoriatic arthritis and patients with psoriatic arthritis, Patrick et al. [37] identified significant loci overlapping the regulatory elements that encompass genes that are differentially expressed in differentiated osteoblasts, including genes that participate in Wnt signaling, such as *RUNX1*, *FUT8*, and *CTNNAL1*. The proteins in Wnt/β-catenin signaling play essential roles in the development of SpA. Xie et al. [38] found that Wnt proteins were essential in normal bone homeostasis, particularly in osteoblastic new bone formation. Therefore, Wnt proteins may also play roles in new bone formation in ankylosing spondylitis, and various Wnt signaling molecules were shown to be involved in maintaining bone mass [39]. In summary, these results provide new clues for future studies on the genetic mechanism of *CTNNAL1*.

In our study, GO enrichment analysis and KEGG pathway analysis were also conducted to explore the functions of candidate genes and how they are distributed in SpA. For example, mitochondrion organization (GO: 0007005) was identified by both TWAS analysis and gene expression profiling. Cytochrome c is primarily known for its role in the mitochondria as a key participant in the life-supporting function of ATP synthesis [40]. Recently, researchers found that cytochrome c can interact with protease, which leads to the activation of the apoptosis protease activation factor [41]. It has been demonstrated that this biological signal is responsible for apoptosis and activation of the inflammatory process during the pathogenesis of psoriatic arthritis [41]. Overall, these findings suggested that abnormal mitochondrial organization may play a role in the pathogenesis of SpA.

Axon guidance (KEGG: hsa04360) was also identified as enriched in SpA. Recently, the semaphorin family was originally identified as axonal guidance molecules, and semaphorins affect the pathogenesis of multiple types of arthritis by regulating immunity, angiogenesis, bone remodeling, apoptosis, cell migration and invasion [42, 43]. In addition, the semaphorin family can regulate the biological pathway of *TNF-α/ADAMTS-4*, and blocking semaphorins can decrease cartilage and bone destruction, cell infiltration into the synovium, and the production of *TNF-α* and *IL-6* [44]. The TWAS analysis and gene expression profile of SpA identified axon guidance as a susceptibility pathway associated with SpA, which was consistent with existing research.

In addition, we found that some common genes identified in skeletal muscle and whole blood tissue showed different risk directions. For example, the Z values of *GGTA1P* in skeletal muscle and whole blood were opposite each other. This difference potentially can be explained in two aspects. First, they may be attributed to the biological variations in gene expression across different tissue types. For instance, studies have reported that in patients with type 2 diabetes, GLUT4 is selectively downregulated only in adipose tissue, while its expression level remains relatively high in skeletal muscle [45]. This also explains why our TWAS results (sampled from skeletal muscle and peripheral blood) do not perfectly overlap with the expression profile results (sampled from intervertebral discs). Second, it has been reported that TWAS exhibits bias in expression profiles from non-trait-related tissues [13]. Therefore, further investigation of the functional roles and mechanisms of these genes across different tissue types is necessary to better explain these disparities.

After strict Bonferroni correction, the significance threshold of the TWAS results was *P* < 5.38 × 10^−6^. *P* values between *P* < 5.38 × 10^−6^ and 0.05 were considered suggestive of significance. Unfortunately, according to our results, the TWAS results showed only suggestive associations with SpA. Because reference panel size affects the *P* value. We should focus more on effective size (*Z* score) instead of *P* value. Although *P* < 5.38 × 10^−6^ and 0.05 were considered to be suggestive of significance, these genes are still valuable.

One of the strengths of TWAS lies in its ability to accurately prioritize genes likely to be causal while excluding non-causal genes [13]. Our study aimed to enhance the accuracy of our results through three approaches. Firstly, we used multi-tissues TWAS in order to produce more accurate results than single-tissue counterparts [18]. Secondly, we performed TWAS analysis based on the latest GWAS summary data for SpA. The large sample size of the GWAS summary data ensured the precision of our research findings. Finally, we validated the candidate genes by comparing them with the gene expression profile, which substantially improved the credibility of our TWAS analysis results.

This study also has some limitations. First, the GWAS summary data are based on those with European ancestry and may not apply to other ancestry studies. Therefore, caution should be taken when applying our results to other populations. Further TWAS analysis of other populations is needed to verify our results. Second, to validate the TWAS results, we compared the significant genes identified by TWAS analysis of SpA with the gene expression profile of jSpA, but jSpA is one subtype of SpA. Our results should be interpreted with caution. Further biological studies should be conducted to confirm our findings.

## Conclusions

In summary, based on GWAS summary data, a TWAS analysis identified novel and common susceptibility genes for SPA. This study not only provides novel clues for understanding the genetic mechanism of SpA but also provides a basis for further experiments. In addition, beyond specific mechanistic findings for SpA, this work outlines a systematic approach for identifying functional mediators of complex-trait diseases.

### Supplementary Information

**Additional file 1:** Risk Loci in GWAS Dataset of SpA.

**Additional file 2:** Quality Control Steps.

**Additional file 3: Table S1** Top genes selected by transcriptome-wide association study (TWAS) analysis

**Additional file 4: Table S2** GO terms identified by Metascape enriched for SpA

## Data Availability

The datasets analyzed during the current study are available from the Gene Expression Omnibus database (https://www.ncbi.nlm.nih.gov/gds) accession number: GSE58667. The GWAS summary data of SpA obtained from UK biobank (https://nealelab.github.io/UKBB_ldsc/downloads.html).

## Abbreviation

DEG: Differentially expressed genes
eQTL: Expression quantitative trait loci
FUSION: Functional summary-based imputation
FUMA: Functional Mapping and Annotation of Genome-wide Association Study
GEO: Gene expression omnibus
GO: Gene ontology
GWAS: Genome-wide association study
KEGG: Kyoto Encyclopedia of Genes and Genomes
TWAS: Transcriptome-wide association study
SNP: Single nucleotide polymorphisms
SpA: Spondyloarthritis
