# Supplementary material for: Transcriptome-wide association study identifies new susceptibility genes and pathways for spondyloarthritis
Source: J Orthop Surg Res. 2023 Sep 4;18:659. doi: 10.1186/s13018-023-04029-4 (PMC10478464; doi:10.1186/s13018-023-04029-4)
Supplement: Supplementary file 2 — Additional file 2: Quality Control Steps. [file 13018_2023_4029_MOESM2_ESM.docx]

**Quality Control Steps**

The GWAS dataset excluded individuals who were identified by the UK Biobank as outliers based on either genotyping missingness rate or heterogeneity, whose sex inferred from the genotypes did not match their self-reported sex and who were not of white ancestry (based on both, self-reported ethnicity and those from whom one of the two first genomic principal components did not fall within 5 standard deviations from the mean). Finally, the GWAS dataset removed individuals with a missingness >5% across variants which passed our quality control procedure and those that have a missing phenotype for 40 or more traits. The resulting study cohort comprised 452,264 individuals.

From the genotyped data, the GWAS dataset only retained bi-allelic autosomal variants which were assayed by both genotyping arrays employed by UK Biobank. The GWAS dataset furthermore excluded variants which had failed UK Biobank quality control procedures in any of the genotyping batches. Additionally, for imputed and genotyped variants, the GWAS dataset excluded variants with P < 10-50 for departure from Hardy-Weinberg, computed on a subset of 344,057 unrelated (Kinship coefficient < 0.0442) individuals in the White-British subset of the study cohort, and with a missingness rate > 2% in the study cohort. Although the GWAS dataset analysed all imputed variants and all genotyped variants with MAF > 10-4 (all results available on the GeneATLAS website), only imputed variants with MAF>10-3 in the study cohort and imputation score larger than 0.9 were used for the summary results presented here. This cut-off corresponds to less than 905 occurrences of the minor allele in the study cohort. The GWAS dataset also filtered the HLA imputed alleles that were present in fewer than 10 individuals.
