# Supplementary material for: Transcriptome-wide association study identifies new susceptibility genes and pathways for spondyloarthritis
Source: J Orthop Surg Res. 2023 Sep 4;18:659. doi: 10.1186/s13018-023-04029-4 (PMC10478464; doi:10.1186/s13018-023-04029-4)
Supplement: Supplementary file 3 — Additional file 3: Table S1 Top genes selected by transcriptome-wide association study (TWAS) analysis [file 13018_2023_4029_MOESM3_ESM.docx]

**Supplementary Table 1. Top genes selected by transcriptome-wide association study (TWAS) analysis**

| Tissue | Gene | CHR | BEST.GWAS.ID | NSNP | TWAS.Z | TWAS.P |
| --- | --- | --- | --- | --- | --- | --- |
| Muscle  Skeletal | NFKBIB | 19 | rs751941 | 397 | 4.0058 | 6.18E-05 |
|  | PGAP1 | 2 | rs2438229 | 348 | -3.85 | 0.000118 |
|  | MCFD2 | 2 | rs6713911 | 556 | 3.829088 | 0.000129 |
|  | CCT3 | 1 | rs35478936 | 411 | 3.6878 | 0.000226 |
|  | AC079776.7 | 2 | rs6705721 | 257 | -3.409347 | 0.000651 |
|  | SEMA5B | 3 | rs1348595 | 485 | -3.406215 | 0.000659 |
|  | GPR108 | 19 | rs10424498 | 475 | -3.355849 | 0.000791 |
|  | RSU1 | 10 | rs921743 | 652 | -3.305839 | 0.000947 |
|  | C2 | 6 | rs4151669 | 227 | 3.262262 | 0.00111 |
|  | ZNF215 | 11 | rs11041157 | 651 | 3.2538 | 0.00114 |
|  | EID1 | 15 | rs784409 | 351 | -3.23628 | 0.00121 |
|  | RAB6C | 2 | rs6705721 | 257 | -3.230948 | 0.001234 |
|  | RP11-473M20.9 | 16 | rs12917910 | 381 | -3.18367 | 0.00145 |
|  | SAMD12 | 8 | rs17683401 | 553 | -3.17728 | 0.00149 |
|  | 2-Mar | 19 | rs17160489 | 352 | -3.1486 | 0.00164 |
|  | SAMD9L | 7 | rs3802062 | 351 | -3.1399 | 0.00169 |
|  | PPM1B | 2 | rs10514791 | 501 | 3.122926 | 0.001791 |
|  | SPRN | 10 | rs2265638 | 282 | 3.112108 | 0.001858 |
|  | TEAD2 | 19 | rs254660 | 374 | 3.0795 | 0.00207 |
|  | ZC3H3 | 8 | rs380904 | 360 | 3.0619 | 0.0022 |
|  | ARTN | 1 | rs37458 | 413 | 3.01359 | 0.002582 |
|  | BEST3 | 12 | rs710711 | 559 | -3.01201 | 0.0026 |
|  | HACE1 | 6 | rs9404586 | 372 | 3.005293 | 0.00265 |
|  | FMO5 | 1 | rs2352866 | 407 | 2.99023 | 0.002788 |
|  | CNNM3 | 2 | rs11694296 | 172 | 2.98343 | 0.00285 |
|  | MVB12B | 9 | rs4837090 | 494 | -2.98241 | 0.00286 |
|  | NLRC3 | 16 | rs12933120 | 363 | 2.98035 | 0.00288 |
|  | RP11-473M20.14 | 16 | rs12917910 | 390 | 2.9747 | 0.00293 |
|  | ZNF100 | 19 | rs1520068 | 298 | 2.969896 | 0.00298 |
|  | TYMS | 18 | rs4798777 | 622 | 2.9589 | 0.00309 |
|  | JMJD7 | 15 | rs316622 | 410 | -2.93012 | 0.00339 |
|  | RP11-7O11.3 | 1 | rs37458 | 409 | -2.92034 | 0.003496 |
|  | HLA-B | 6 | rs2523590 | 219 | 2.912403 | 0.00359 |
|  | MRPS36 | 5 | rs2932770 | 289 | 2.9045 | 0.00368 |
|  | ABCC3 | 17 | rs28498091 | 436 | -2.90325 | 0.00369 |
|  | ZNF493 | 19 | rs1520068 | 286 | 2.8573 | 0.00427 |
|  | ZNF429 | 19 | rs1520068 | 299 | 2.8573 | 0.00427 |
|  | BTF3 | 5 | rs4515275 | 532 | -2.8567 | 0.00428 |
|  | RP11-420K14.1 | 19 | rs1520068 | 314 | 2.8464 | 0.00442 |
|  | APOPT1 | 14 | rs3212042 | 361 | -2.84 | 0.00452 |
|  | LILRA2 | 19 | rs4806637 | 489 | -2.831 | 0.00464 |
|  | JPH2 | 20 | rs6031697 | 501 | 2.831 | 0.00464 |
|  | CLUAP1 | 16 | rs12917910 | 366 | -2.8303 | 0.00465 |
|  | ZNF43 | 19 | rs1520068 | 294 | 2.825167 | 0.00473 |
|  | PRKAB2 | 1 | rs17355509 | 380 | 2.81065 | 0.004944 |
|  | CDK7 | 5 | rs2932770 | 280 | 2.8016 | 0.00508 |
|  | FBXO40 | 3 | rs4678186 | 341 | -2.792494 | 0.00523 |
|  | EMC9 | 14 | rs2277479 | 519 | 2.78 | 0.0054 |
|  | LYRM9 | 17 | rs16966724 | 312 | 2.78159 | 0.00541 |
|  | THSD7A | 7 | rs2077983 | 688 | 2.7719 | 0.00557 |
|  | AL020996.1 | 1 | rs213637 | 377 | 2.7702 | 0.005602 |
|  | MPDZ | 9 | rs10491741 | 515 | 2.76643 | 0.00567 |
|  | C1QL3 | 10 | rs921743 | 602 | 2.749241 | 0.005973 |
|  | FHL2 | 2 | rs7604966 | 597 | 2.737439 | 0.006192 |
|  | CEP104 | 1 | rs11589102 | 454 | -2.73367 | 0.006263 |
|  | KLHL12 | 1 | rs6699298 | 490 | -2.72696 | 0.006392 |
|  | ZCCHC3 | 20 | rs6037986 | 428 | -2.71766 | 0.00657 |
|  | LINC00664 | 19 | rs1520068 | 295 | 2.714835 | 0.00663 |
|  | DCAF16 | 4 | rs13130097 | 380 | -2.71505 | 0.00663 |
|  | ADH4 | 4 | rs6532811 | 498 | -2.71466 | 0.00663 |
|  | TIPARP | 3 | rs9840556 | 433 | -2.708266 | 0.006764 |
|  | RP5-1027O11.1 | 1 | rs4233325 | 451 | -2.70576 | 0.006815 |
|  | C6orf201 | 6 | rs9378811 | 637 | -2.694792 | 0.00704 |
|  | RP11-111A22.1 | 15 | rs2289328 | 368 | -2.694 | 0.00706 |
|  | CMYA5 | 5 | rs6453450 | 450 | -2.68873 | 0.00717 |
|  | RASA4 | 7 | rs2734605 | 214 | -2.68521 | 0.00725 |
|  | LUZPP1 | 3 | rs3827494 | 381 | -2.66 | 0.007814 |
|  | GGTA1P | 9 | rs177698 | 442 | 2.65871 | 0.00784 |
|  | Z97634.3 | 16 | rs4984867 | 385 | 2.6432 | 0.00821 |
|  | ITGB1BP1 | 2 | rs10495570 | 433 | -2.637352 | 0.008356 |
|  | BAG5 | 14 | rs3212042 | 361 | -2.63 | 0.00848 |
|  | CCHCR1 | 6 | rs2523590 | 176 | -2.627424 | 0.0086 |
|  | RP11-678G14.4 | 19 | rs1520068 | 307 | 2.621347 | 0.00876 |
|  | ALMS1P | 2 | rs17008862 | 356 | 2.620246 | 0.008787 |
|  | HIBADH | 7 | rs1404282 | 487 | 2.61633 | 0.00889 |
|  | ANKK1 | 11 | rs12365214 | 518 | -2.61489 | 0.00893 |
|  | MASP1 | 3 | rs17366653 | 599 | 2.6007 | 0.009303 |
|  | CASP16 | 16 | rs12917910 | 392 | -2.5961 | 0.00943 |
|  | GTPBP4 | 10 | rs2246654 | 502 | -2.592811 | 0.00952 |
|  | GSDMD | 8 | rs380904 | 357 | 2.58787 | 0.00966 |
|  | DISP2 | 15 | rs2289328 | 399 | 2.58101 | 0.00985 |
|  | MFI2 | 3 | rs11717211 | 478 | -2.578916 | 0.009911 |
|  | FAM19A5 | 22 | rs742025 | 793 | -2.5714 | 0.0101 |
|  | CHD1L | 1 | rs2352866 | 407 | -2.56224 | 0.0104 |
|  | RP11-678G14.3 | 19 | rs1520068 | 307 | 2.560227 | 0.0105 |
|  | FRMD3 | 9 | rs6559711 | 489 | 2.5585 | 0.01051 |
|  | LRRK1 | 15 | rs8042424 | 627 | -2.55564 | 0.0106 |
|  | HOXA5 | 7 | rs1404282 | 396 | 2.55413 | 0.01065 |
|  | RP11-545I5.3 | 6 | rs397311 | 265 | -2.553994 | 0.01065 |
|  | ANKLE2 | 12 | rs1278603 | 250 | 2.5523 | 0.0107 |
|  | AIM2 | 1 | rs16841682 | 472 | -2.54994 | 0.010774 |
|  | SGCB | 4 | rs2271045 | 199 | -2.54903 | 0.0108 |
|  | CTD-2555K7.2 | 14 | rs8019439 | 607 | 2.54 | 0.01097 |
|  | DKFZP761J1410 | 19 | rs34092 | 398 | -2.544267 | 0.011 |
|  | RP11-73M18.6 | 14 | rs3759579 | 352 | 2.54 | 0.01109 |
|  | TRPV2 | 17 | rs4570894 | 309 | -2.5373 | 0.01117 |
|  | RP11-314C16.1 | 6 | rs2327142 | 400 | 2.530394 | 0.01139 |
|  | ZDHHC8 | 22 | rs1139793 | 415 | 2.531 | 0.0114 |
|  | FRYL | 4 | rs11730515 | 225 | 2.52586 | 0.01154 |
|  | MCRS1 | 12 | rs836968 | 324 | 2.5226 | 0.0116 |
|  | ABCA6 | 17 | rs7212506 | 467 | 2.5232 | 0.01163 |
|  | AC000078.5 | 22 | rs1139793 | 551 | -2.5198 | 0.0117 |
|  | TRIM25 | 17 | rs792379 | 465 | -2.5103 | 0.01206 |
|  | STX7 | 6 | rs8192627 | 551 | 2.501674 | 0.01236 |
|  | SEMA4A | 1 | rs35478936 | 353 | -2.49113 | 0.012734 |
|  | SYCP2L | 6 | rs1225763 | 474 | 2.488371 | 0.01283 |
|  | SLC25A16 | 10 | rs1136645 | 403 | -2.482928 | 0.013031 |
|  | BUD31 | 7 | rs10282706 | 309 | 2.47938 | 0.01316 |
|  | LYRM2 | 6 | rs9444682 | 492 | -2.476056 | 0.01328 |
|  | RP11-517C16.2 | 16 | rs8063973 | 897 | 2.47207 | 0.01343 |
|  | DNA2 | 10 | rs1136645 | 384 | 2.47201 | 0.013436 |
|  | BID | 22 | rs2895951 | 530 | -2.465 | 0.0137 |
|  | AC005224.2 | 17 | rs16949319 | 635 | -2.46364 | 0.01375 |
|  | SPDYE2 | 7 | rs2734605 | 235 | -2.4635 | 0.01376 |
|  | PDPK2 | 16 | rs12917910 | 308 | -2.46042 | 0.01388 |
|  | ABCC5 | 3 | rs11706273 | 483 | -2.460245 | 0.013884 |
|  | PAOX | 10 | rs2265638 | 348 | -2.45704 | 0.014009 |
|  | SMYD1 | 2 | rs7425197 | 243 | -2.456564 | 0.014027 |
|  | BHMT | 5 | rs6453450 | 416 | -2.45251 | 0.01419 |
|  | REPIN1 | 7 | rs11772167 | 373 | 2.44935 | 0.01431 |
|  | GATSL3 | 22 | rs17729168 | 426 | 2.4473 | 0.0144 |
|  | WDR1 | 4 | rs4235358 | 596 | -2.44301 | 0.01457 |
|  | NUDCD3 | 7 | rs10230538 | 352 | -2.44064 | 0.01466 |
|  | TNP1 | 2 | rs1111341 | 514 | 2.437954 | 0.014771 |
|  | RP11-254F7.2 | 2 | rs10495570 | 579 | -2.435898 | 0.014855 |
|  | KRT32 | 17 | rs11653427 | 455 | 2.43494 | 0.01489 |
|  | RAB3IP | 12 | rs710711 | 561 | -2.42927 | 0.0151 |
|  | RPRD1B | 20 | rs6022123 | 482 | -2.4265 | 0.01525 |
|  | KHDC1 | 6 | rs3915865 | 394 | -2.424383 | 0.01533 |
|  | RPAP1 | 15 | rs316622 | 364 | 2.4213 | 0.01547 |
|  | CDYL | 6 | rs200852 | 664 | -2.420172 | 0.01551 |
|  | MTFR1L | 1 | rs213637 | 375 | 2.41541 | 0.015717 |
|  | NTNG2 | 9 | rs10901089 | 484 | 2.41275 | 0.01583 |
|  | RP11-611E13.2 | 12 | rs10506581 | 575 | 2.4076 | 0.0161 |
|  | UFSP2 | 4 | rs2310357 | 551 | 2.40237 | 0.01629 |
|  | PHRF1 | 11 | rs6598023 | 450 | 2.39826 | 0.01647 |
|  | PDE4C | 19 | rs4808100 | 412 | -2.397372 | 0.0165 |
|  | B4GALT2 | 1 | rs37458 | 412 | -2.39201 | 0.016756 |
|  | CACUL1 | 10 | rs7914808 | 506 | -2.391964 | 0.016758 |
|  | RP11-23P13.7 | 15 | rs316622 | 424 | -2.39073 | 0.01681 |
|  | RP11-678G14.2 | 19 | rs1520068 | 306 | 2.38878 | 0.0169 |
|  | RMDN3 | 15 | rs2289328 | 309 | 2.38432 | 0.01711 |
|  | CCDC74B | 2 | rs6705721 | 187 | -2.384344 | 0.01711 |
|  | PHOSPHO2 | 2 | rs2544374 | 467 | -2.382605 | 0.017191 |
|  | ANAPC4 | 4 | rs316776 | 542 | 2.38153 | 0.01724 |
|  | NCK2 | 2 | rs7604966 | 633 | -2.3806 | 0.017284 |
|  | TTC32 | 2 | rs1427538 | 591 | 2.379816 | 0.017321 |
|  | BAHD1 | 15 | rs2289328 | 387 | 2.3755 | 0.01753 |
|  | B3GNT9 | 16 | rs749242 | 269 | -2.37436 | 0.01758 |
|  | PDE6G | 17 | rs9911383 | 326 | -2.37377 | 0.01761 |
|  | WDR12 | 2 | rs2255047 | 262 | -2.37321 | 0.017634 |
|  | NNT | 5 | rs12655504 | 254 | 2.37122 | 0.01773 |
|  | EXOSC5 | 19 | rs1046909 | 375 | 2.369118 | 0.0178 |
|  | SRP19 | 5 | rs10463643 | 465 | -2.3658 | 0.01799 |
|  | CTD-3092A11.1 | 15 | rs16956362 | 133 | 2.36423 | 0.01807 |
|  | NUP43 | 6 | rs9397708 | 457 | 2.362724 | 0.01814 |
|  | CYB5A | 18 | rs17807682 | 647 | 2.3589 | 0.01833 |
|  | RP11-165J3.6 | 9 | rs10512225 | 493 | -2.35321 | 0.01861 |
|  | TMEM178A | 2 | rs6716299 | 442 | 2.351152 | 0.018715 |
|  | ERAL1 | 17 | rs2286519 | 318 | 2.3486 | 0.01884 |
|  | CTB-75G16.3 | 17 | rs295839 | 317 | -2.34794 | 0.01888 |
|  | COMMD6 | 13 | rs1108412 | 556 | 2.347 | 0.0189 |
|  | MRPL40P1 | 12 | rs1316645 | 531 | -2.34474 | 0.019 |
|  | RAF1 | 3 | rs165269 | 514 | 2.337625 | 0.019407 |
|  | MTG1 | 10 | rs2265638 | 342 | 2.337169 | 0.01943 |
|  | RP11-337C18.8 | 1 | rs17355509 | 380 | -2.3366 | 0.01946 |
|  | HLA-DQB2 | 6 | rs2269346 | 254 | 2.331056 | 0.01975 |
|  | LARS | 5 | rs17104772 | 433 | -2.3304 | 0.01978 |
|  | TMEM101 | 17 | rs1731902 | 363 | -2.3299 | 0.01981 |
|  | AP000442.1 | 11 | rs35211634 | 342 | 2.32044 | 0.02032 |
|  | NUDT9 | 4 | rs17012830 | 459 | 2.31823 | 0.02044 |
|  | C17orf49 | 17 | rs312470 | 504 | -2.3112 | 0.02082 |
|  | MROH5 | 8 | rs6987971 | 473 | -2.3107 | 0.02085 |
|  | LMO7 | 13 | rs1108412 | 557 | 2.3071 | 0.021 |
|  | NT5C3B | 17 | rs11653427 | 409 | -2.3002 | 0.02144 |
|  | PITX3 | 10 | rs10748816 | 297 | -2.297 | 0.021619 |
|  | ZNF738 | 19 | rs1520068 | 285 | 2.296074 | 0.0217 |
|  | CTA-390C10.10 | 22 | rs7292626 | 520 | -2.2909 | 0.022 |
|  | SLC22A1 | 6 | rs3822841 | 544 | -2.286167 | 0.02224 |
|  | SPTBN5 | 15 | rs316622 | 418 | -2.28604 | 0.02225 |
|  | RP3-414A15.10 | 14 | rs7523 | 385 | -2.28 | 0.02276 |
|  | CTBP1-AS2 | 4 | rs4865438 | 404 | -2.2683 | 0.02331 |
|  | ROBO3 | 11 | rs4078313 | 497 | -2.26408 | 0.02357 |
|  | ADRBK2 | 22 | rs7292626 | 579 | -2.2611 | 0.0238 |
|  | FAM134C | 17 | rs10454087 | 276 | 2.2604 | 0.0238 |
|  | DMWD | 19 | rs34014819 | 400 | 2.25796 | 0.0239 |
|  | FAM169A | 5 | rs6453084 | 454 | -2.25714 | 0.024 |
|  | DPH2 | 1 | rs37458 | 412 | 2.2543 | 0.024177 |
|  | RUSC1-AS1 | 1 | rs951241 | 264 | -2.2537 | 0.024215 |
|  | EXTL1 | 1 | rs213637 | 446 | 2.25331 | 0.02424 |
|  | CCDC125 | 5 | rs2932770 | 235 | -2.24498 | 0.02477 |
|  | FILIP1 | 6 | rs6929742 | 318 | -2.2447 | 0.02479 |
|  | RP11-20I23.13 | 16 | rs8054693 | 291 | 2.24254 | 0.02493 |
|  | EMR4P | 19 | rs10424498 | 422 | 2.2397 | 0.0251 |
|  | NAF1 | 4 | rs10029699 | 372 | -2.23977 | 0.02511 |
|  | YWHAZ | 8 | rs7815210 | 482 | -2.23549 | 0.02539 |
|  | SRR | 17 | rs1885987 | 409 | -2.23538 | 0.02539 |
|  | PCMTD1 | 8 | rs890328 | 428 | -2.23293 | 0.02555 |
|  | ZNF611 | 19 | rs34312893 | 454 | 2.23 | 0.0257 |
|  | POLR2J3 | 7 | rs2734605 | 228 | -2.22852 | 0.02585 |
|  | HSD17B7P2 | 10 | rs2505192 | 138 | -2.224704 | 0.026101 |
|  | FAM179A | 2 | rs877832 | 596 | 2.224 | 0.026148 |
|  | DSTN | 20 | rs6034809 | 653 | -2.22365 | 0.02617 |
|  | ZNF264 | 19 | rs917340 | 509 | 2.2215 | 0.0263 |
|  | COX7A1 | 19 | rs2112971 | 334 | 2.221718 | 0.0263 |
|  | ALDH7A1 | 5 | rs1051643 | 416 | 2.22006 | 0.02641 |
|  | CTSH | 15 | rs11636732 | 436 | -2.21909 | 0.02648 |
|  | ENOSF1 | 18 | rs4798777 | 626 | 2.2189 | 0.02649 |
|  | GOT2 | 16 | rs12447862 | 459 | 2.21582 | 0.0267 |
|  | CYB5R4 | 6 | rs3778203 | 345 | 2.2135 | 0.02686 |
|  | Metazoa_SRP | 21 | rs2839274 | 337 | -2.2083 | 0.0272 |
|  | PCNT | 21 | rs2839274 | 454 | 2.20717 | 0.0273 |
|  | CCDC127 | 5 | rs11745733 | 290 | -2.20623 | 0.02737 |
|  | TMCC2 | 1 | rs10900461 | 588 | -2.20049 | 0.027772 |
|  | RP11-486A14.2 | 12 | rs11107199 | 410 | 2.20037 | 0.0278 |
|  | C19orf69 | 19 | rs1046909 | 362 | -2.1999 | 0.0278 |
|  | SND1 | 7 | rs2237798 | 458 | 2.19976 | 0.02782 |
|  | DMGDH | 5 | rs6453450 | 423 | -2.19973 | 0.02783 |
|  | G6PC3 | 17 | rs1731902 | 361 | 2.19917 | 0.02787 |
|  | RP11-23P13.6 | 15 | rs316622 | 421 | -2.19882 | 0.02789 |
|  | HHIPL1 | 14 | rs12586338 | 447 | 2.2 | 0.0279 |
|  | MBOAT7 | 19 | rs17836409 | 475 | -2.195109 | 0.0282 |
|  | C18orf8 | 18 | rs12373268 | 346 | -2.1939 | 0.02824 |
|  | SNX24 | 5 | rs13167121 | 449 | 2.1939 | 0.02824 |
|  | RP11-509J21.4 | 9 | rs6476798 | 645 | 2.1905 | 0.02849 |
|  | TGM2 | 20 | rs6022123 | 472 | -2.19011 | 0.02852 |
|  | SCARB2 | 4 | rs17002074 | 484 | -2.1881 | 0.02866 |
|  | TRAF3IP2 | 6 | rs6933627 | 438 | 2.187402 | 0.02871 |
|  | CECR5 | 22 | rs5748966 | 517 | -2.1864 | 0.0288 |
|  | ARFGAP3 | 22 | rs2179258 | 531 | 2.1865 | 0.0288 |
|  | BNIP1 | 5 | rs3797456 | 569 | -2.1862 | 0.0288 |
|  | PRTG | 15 | rs11071185 | 393 | 2.18415 | 0.02895 |
|  | CLEC3B | 3 | rs1915084 | 431 | -2.182758 | 0.029054 |
|  | ANKRD39 | 2 | rs11694296 | 169 | 2.182549 | 0.029069 |
|  | RPL26L1 | 5 | rs10053713 | 538 | 2.1825 | 0.02907 |
|  | HOMER1 | 5 | rs6453450 | 438 | -2.17806 | 0.0294 |
|  | SOCS1 | 16 | rs3931016 | 513 | -2.17356 | 0.02974 |
|  | GPR179 | 17 | rs7211741 | 298 | -2.17128 | 0.02991 |
|  | MRPS14 | 1 | rs859460 | 442 | -2.17069 | 0.029954 |
|  | HRAS | 11 | rs6598023 | 430 | -2.16951 | 0.03004 |
|  | STK17B | 2 | rs7575378 | 371 | -2.164765 | 0.030406 |
|  | CTNNAL1 | 9 | rs1319977 | 622 | 2.16453 | 0.03042 |
|  | KDELR2 | 7 | rs11764399 | 306 | 2.1631 | 0.03053 |
|  | LONP1 | 19 | rs778988 | 400 | 2.160027 | 0.0308 |
|  | SLC7A9 | 19 | rs8102279 | 421 | 2.155485 | 0.0311 |
|  | FBXL4 | 6 | rs9492351 | 454 | 2.154203 | 0.03122 |
|  | RP11-108P20.1 | 18 | rs8091691 | 579 | 2.1532 | 0.0313 |
|  | CAPG | 2 | rs17761664 | 459 | 2.151953 | 0.031401 |
|  | KCNC4 | 1 | rs7527452 | 567 | 2.15022 | 0.031538 |
|  | PKIB | 6 | rs9375122 | 437 | -2.147045 | 0.03179 |
|  | METAP1D | 2 | rs10930497 | 474 | -2.145975 | 0.031875 |
|  | LRPPRC | 2 | rs10514791 | 507 | -2.144469 | 0.031995 |
|  | CTD-2561J22.5 | 19 | rs1520068 | 299 | 2.142024 | 0.0322 |
|  | TOMM70A | 3 | rs11915096 | 403 | 2.136897 | 0.032606 |
|  | USP1 | 1 | rs10889320 | 449 | -2.1351 | 0.032753 |
|  | SEPN1 | 1 | rs213637 | 365 | -2.13211 | 0.032998 |
|  | RAPGEFL1 | 17 | rs2269457 | 295 | -2.12755 | 0.03337 |
|  | FNDC5 | 1 | rs194663 | 274 | -2.12658 | 0.033455 |
|  | TCFL5 | 20 | rs3765462 | 481 | 2.1257 | 0.03353 |
|  | AEBP1 | 7 | rs10230538 | 354 | 2.12562 | 0.03354 |
|  | MCM4 | 8 | rs11786911 | 188 | -2.1232 | 0.03374 |
|  | IGFBP2 | 2 | rs6728666 | 529 | -2.122957 | 0.033757 |
|  | BHMT2 | 5 | rs6453450 | 419 | -2.11991 | 0.03401 |
|  | MAN1A2 | 1 | rs6698100 | 485 | -2.1184 | 0.034141 |
|  | NOMO1 | 16 | rs7404450 | 123 | 2.11624 | 0.03432 |
|  | AC013439.4 | 2 | rs12466395 | 301 | 2.1148 | 0.034447 |
|  | FBLN7 | 2 | rs4299347 | 350 | -2.114444 | 0.034477 |
|  | RARRES2 | 7 | rs11772167 | 353 | 2.11067 | 0.0348 |
|  | PARD6G | 18 | rs12960174 | 194 | 2.1092 | 0.03492 |
|  | TFB1M | 6 | rs11752914 | 518 | -2.108485 | 0.03499 |
|  | CTD-3064M3.7 | 8 | rs6987971 | 473 | -2.10689 | 0.03513 |
|  | PLAC8L1 | 5 | rs17104772 | 412 | 2.10667 | 0.03515 |
|  | PRIMPOL | 4 | rs12513029 | 576 | 2.10553 | 0.03524 |
|  | HEATR2 | 7 | rs28448690 | 294 | 2.1052 | 0.03527 |
|  | PADI4 | 1 | rs2240335 | 462 | -2.10357 | 0.035416 |
|  | REEP5 | 5 | rs10463643 | 494 | 2.0989 | 0.03583 |
|  | RP11-227G15.2 | 17 | rs6505306 | 262 | 2.09312 | 0.03634 |
|  | KLC1 | 14 | rs3212042 | 361 | -2.09 | 0.03638 |
|  | HLA-DQA1 | 6 | rs17429444 | 214 | -2.089624 | 0.03665 |
|  | LIMA1 | 12 | rs836968 | 315 | -2.0871 | 0.0369 |
|  | RP11-218M22.1 | 12 | rs8181744 | 457 | -2.08525 | 0.037 |
|  | CCL28 | 5 | rs12655504 | 289 | -2.08564 | 0.03701 |
|  | PNMA1 | 14 | rs7523 | 405 | 2.08 | 0.0371 |
|  | FAM78A | 9 | rs10901089 | 489 | -2.08445 | 0.03712 |
|  | KIAA1109 | 4 | rs1383228 | 318 | 2.0843 | 0.03713 |
|  | GUSBP3 | 5 | rs2932770 | 99 | -2.08213 | 0.03733 |
|  | GNB1L | 22 | rs1139793 | 567 | -2.0817 | 0.0374 |
|  | RP11-867G23.1 | 11 | rs17582367 | 325 | 2.08014 | 0.03751 |
|  | PKM | 15 | rs12441929 | 295 | -2.07814 | 0.0377 |
|  | C11orf74 | 11 | rs12419135 | 582 | -2.07729 | 0.03778 |
|  | DUSP13 | 10 | rs1259498 | 316 | -2.076429 | 0.037854 |
|  | CCDC158 | 4 | rs17002074 | 520 | -2.07557 | 0.03793 |
|  | CLIC5 | 6 | rs17216646 | 467 | -2.0732 | 0.03815 |
|  | WNT5B | 12 | rs8181744 | 451 | 2.07177 | 0.0383 |
|  | RGPD8 | 2 | rs4299347 | 404 | -2.070507 | 0.038405 |
|  | PNPT1 | 2 | rs10165864 | 450 | 2.070131 | 0.03844 |
|  | ZNF205 | 16 | rs12917910 | 385 | -2.06861 | 0.03858 |
|  | MBLAC1 | 7 | rs3735453 | 281 | -2.0665 | 0.03878 |
|  | CNNM4 | 2 | rs11694296 | 183 | -2.0655 | 0.038876 |
|  | YME1L1 | 10 | rs2800393 | 457 | -2.065004 | 0.038923 |
|  | RP11-96C23.11 | 10 | rs4934243 | 243 | 2.063507 | 0.039065 |
|  | IL18R1 | 2 | rs1997502 | 572 | -2.061736 | 0.039233 |
|  | HRASLS | 3 | rs9851984 | 410 | -2.059545 | 0.039442 |
|  | AC091729.9 | 7 | rs28448690 | 400 | 2.05951 | 0.03945 |
|  | ATP5SL | 19 | rs1046909 | 363 | -2.054843 | 0.0399 |
|  | PGAP3 | 17 | rs2269457 | 318 | 2.05473 | 0.03991 |
|  | APOB | 2 | rs7576149 | 425 | 2.052261 | 0.040144 |
|  | MUT | 6 | rs9473583 | 347 | 2.051224 | 0.04025 |
|  | KATNA1 | 6 | rs4870303 | 439 | -2.04996 | 0.04037 |
|  | RFPL3S | 22 | rs80272 | 648 | -2.0499 | 0.0404 |
|  | GALNT3 | 2 | rs7577411 | 436 | 2.048529 | 0.040508 |
|  | CWF19L2 | 11 | rs4754221 | 470 | 2.0481 | 0.04055 |
|  | SLC37A1 | 21 | rs13048709 | 725 | -2.0479 | 0.0406 |
|  | NOS2 | 17 | rs16966724 | 304 | -2.0472 | 0.04064 |
|  | FLYWCH1 | 16 | rs12917910 | 340 | -2.04662 | 0.0407 |
|  | CTC-428G20.3 | 5 | rs1005056 | 403 | -2.04344 | 0.04101 |
|  | RP11-250B2.6 | 6 | rs7738642 | 440 | 2.042538 | 0.0411 |
|  | ZNF205-AS1 | 16 | rs12917910 | 388 | 2.04107 | 0.04124 |
|  | NME1-NME2 | 17 | rs7217500 | 443 | 2.03975 | 0.04138 |
|  | C12orf29 | 12 | rs2041859 | 282 | -2.03917 | 0.0414 |
|  | SEPT2 | 2 | rs41342147 | 376 | -2.039412 | 0.041409 |
|  | ERAP1 | 5 | rs10053056 | 501 | -2.03937 | 0.04141 |
|  | RANBP9 | 6 | rs3734669 | 530 | 2.034354 | 0.04192 |
|  | C11orf83 | 11 | rs11568486 | 368 | -2.0322 | 0.04213 |
|  | RP11-2E17.1 | 15 | rs3858964 | 505 | 2.03135 | 0.04222 |
|  | HTR2B | 2 | rs12694884 | 428 | -2.029372 | 0.04242 |
|  | RP11-62F24.2 | 9 | rs7031602 | 621 | -2.02346 | 0.04303 |
|  | ZNF713 | 7 | rs12718975 | 321 | -2.02155 | 0.04322 |
|  | AURKC | 19 | rs917340 | 508 | 2.020486 | 0.0433 |
|  | ATRNL1 | 10 | rs11196869 | 288 | 2.019533 | 0.043432 |
|  | SGTA | 19 | rs308060 | 439 | 2.018976 | 0.0435 |
|  | ST3GAL3 | 1 | rs37458 | 422 | 2.01886 | 0.043502 |
|  | SGOL2 | 2 | rs2293528 | 487 | -2.0167 | 0.043727 |
|  | CNP | 17 | rs11653427 | 374 | 2.01463 | 0.04394 |
|  | FRAS1 | 4 | rs17471104 | 477 | 2.01434 | 0.04397 |
|  | MED16 | 19 | rs11668291 | 480 | 2.014257 | 0.044 |
|  | PDK1 | 2 | rs17581715 | 497 | -2.013657 | 0.044046 |
|  | ZSCAN5A | 19 | rs6509986 | 577 | 2.013202 | 0.0441 |
|  | PLA2R1 | 2 | rs13005826 | 425 | 2.0121 | 0.044209 |
|  | LRRC61 | 7 | rs11772167 | 348 | 2.01211 | 0.04421 |
|  | GPSM1 | 9 | rs7045617 | 369 | -2.0119 | 0.04423 |
|  | PPP1R3A | 7 | rs13222538 | 260 | 2.01103 | 0.04432 |
|  | UBAP2 | 9 | rs10758257 | 314 | -2.01024 | 0.04441 |
|  | FASTKD5 | 20 | rs2740210 | 419 | 2.00904 | 0.04453 |
|  | RP4-800M22.1 | 1 | rs7512076 | 230 | -2.00732 | 0.044716 |
|  | MESDC2 | 15 | rs11072940 | 463 | 2.00731 | 0.04472 |
|  | CABLES2 | 20 | rs3765462 | 487 | -2.00669 | 0.04478 |
|  | RP11-350J20.5 | 6 | rs9397708 | 468 | -2.005258 | 0.04494 |
|  | ATP6V1H | 8 | rs7824834 | 385 | -2.0045 | 0.04502 |
|  | LINC01088 | 4 | rs17471104 | 332 | 2.0038 | 0.04509 |
|  | IDH3A | 15 | rs7168166 | 414 | -2.00308 | 0.04517 |
|  | POLG2 | 17 | rs6504248 | 227 | -2.00124 | 0.04537 |
|  | LMO2 | 11 | rs7123907 | 503 | 2.00073 | 0.04542 |
|  | REEP6 | 19 | rs17604735 | 406 | -1.9998 | 0.0455 |
|  | PIGU | 20 | rs6088514 | 319 | -1.99997 | 0.0455 |
|  | MASTL | 10 | rs2800393 | 457 | -1.9991 | 0.045598 |
|  | AKT1S1 | 19 | rs7258705 | 347 | 1.9968 | 0.0458 |
|  | LL22NC03-N27C7.1 | 22 | rs5996577 | 461 | -1.9962 | 0.0459 |
|  | KCNE3 | 11 | rs11236122 | 414 | 1.9941 | 0.04614 |
|  | RWDD4 | 4 | rs6552675 | 442 | 1.99294 | 0.04627 |
|  | AK4 | 1 | rs7526688 | 446 | 1.9929 | 0.046272 |
|  | TUBG2 | 17 | rs10454087 | 260 | -1.9893 | 0.04667 |
|  | ESPNL | 2 | rs3754703 | 492 | 1.987275 | 0.046892 |
|  | CTB-31O20.4 | 19 | rs17604735 | 384 | 1.9869 | 0.0469 |
|  | CTD-2538C1.2 | 19 | rs8102279 | 404 | -1.987377 | 0.0469 |
|  | TBK1 | 12 | rs10878177 | 317 | 1.9865 | 0.047 |
|  | ZNF10 | 12 | rs1278603 | 164 | -1.9853 | 0.0471 |
|  | NSA2 | 5 | rs6453084 | 476 | -1.98362 | 0.0473 |
|  | ANKS1A | 6 | rs2057537 | 378 | 1.983199 | 0.04735 |
|  | TLCD1 | 17 | rs2286519 | 316 | -1.98123 | 0.04757 |
|  | BGLAP | 1 | rs35478936 | 386 | -1.97857 | 0.047864 |
|  | PSMA7 | 20 | rs944893 | 535 | -1.9762 | 0.04813 |
|  | NACAD | 7 | rs1294889 | 436 | -1.97614 | 0.04814 |
|  | ZFAND2A | 7 | rs28448690 | 401 | 1.97604 | 0.04815 |
|  | KANSL2 | 12 | rs6580689 | 378 | 1.9731 | 0.0485 |
|  | RP11-247A12.7 | 9 | rs10739751 | 399 | 1.97117 | 0.0487 |
|  | RAD17 | 5 | rs2932770 | 227 | 1.9701 | 0.04883 |
|  | RNASEL | 1 | rs10489964 | 456 | 1.97001 | 0.048837 |
|  | PCNX | 14 | rs2189807 | 410 | 1.97 | 0.04898 |
|  | TSFM | 12 | rs7978954 | 355 | 1.96757 | 0.0491 |
|  | VMAC | 19 | rs778988 | 384 | -1.967084 | 0.0492 |
|  | BPHL | 6 | rs913534 | 645 | -1.966888 | 0.0492 |
|  | DNAJC28 | 21 | rs16990664 | 470 | 1.9649 | 0.0494 |
|  | DTX3L | 3 | rs4678186 | 505 | 1.963104 | 0.049634 |
|  | RP11-95O2.5 | 18 | rs1786814 | 376 | -1.963 | 0.04965 |
|  | FAM20A | 17 | rs17701598 | 467 | 1.96263 | 0.04969 |
|  | UBALD1 | 16 | rs231613 | 422 | 1.9622 | 0.04974 |
|  | COQ3 | 6 | rs9389752 | 476 | 1.961815 | 0.04978 |
|  | PRKCSH | 19 | rs34092 | 396 | 1.960894 | 0.0499 |
|  | AMD1 | 6 | rs6933627 | 379 | 1.960117 | 0.04998 |
| Whole  Blood | AC000078.5 | 22 | rs1139793 | 514 | -3.9967 | 6.42E-05 |
|  | RP11-165J3.6 | 9 | rs10512225 | 473 | -3.1153 | 0.00184 |
|  | CTC-301O7.4 | 19 | rs254660 | 358 | -3.0001 | 0.0027 |
|  | ZNF100 | 19 | rs1520068 | 295 | 2.9885 | 0.0028 |
|  | KCNMB4 | 12 | rs10506581 | 609 | -2.9735 | 0.00294 |
|  | GTF2H2C | 5 | rs34221525 | 145 | -2.9619 | 0.00306 |
|  | SLC6A16 | 19 | rs254660 | 370 | -2.9463 | 0.00322 |
|  | GFOD1 | 6 | rs12210311 | 536 | -2.86105 | 0.00422 |
|  | PAQR6 | 1 | rs35478936 | 373 | -2.8549 | 0.00431 |
|  | SMG5 | 1 | rs35478936 | 382 | -2.8549 | 0.00431 |
|  | TMEM79 | 1 | rs35478936 | 379 | -2.8549 | 0.00431 |
|  | C1orf85 | 1 | rs35478936 | 379 | -2.8549 | 0.00431 |
|  | ZNF493 | 19 | rs1520068 | 283 | 2.8464 | 0.00442 |
|  | PPP1R14A | 19 | rs3786870 | 350 | 2.82811 | 0.00468 |
|  | RP11-407N17.5 | 14 | rs4899200 | 365 | -2.7739 | 0.00554 |
|  | DBNDD1 | 16 | rs4785763 | 248 | 2.7617 | 0.00575 |
|  | ZNF429 | 19 | rs1520068 | 304 | 2.73754 | 0.00619 |
|  | STK17B | 2 | rs7575378 | 376 | -2.7341 | 0.00626 |
|  | RP11-122G18.5 | 1 | rs4656334 | 393 | -2.72581 | 0.00641 |
|  | ZC3H3 | 8 | rs380904 | 353 | 2.69647 | 0.00701 |
|  | NUDT16P | 3 | rs10512802 | 405 | -2.68516 | 0.00725 |
|  | ZNF738 | 19 | rs1520068 | 276 | 2.67293 | 0.00752 |
|  | CTD-2265M8.2 | 19 | rs10410302 | 466 | -2.64226 | 0.00824 |
|  | EVI5 | 1 | rs2031494 | 344 | 2.61116 | 0.00902 |
|  | C5orf34 | 5 | rs12655504 | 259 | 2.6074 | 0.00912 |
|  | MAEA | 4 | rs2306242 | 374 | 2.59679 | 0.00941 |
|  | CTNNAL1 | 9 | rs1319977 | 633 | 2.59054 | 0.00958 |
|  | MED27 | 9 | rs10901089 | 592 | 2.5785 | 0.00992 |
|  | HLA-DQB1 | 6 | rs17429444 | 217 | -2.57273 | 0.01009 |
|  | CDK5RAP3 | 17 | rs1912155 | 387 | 2.5693 | 0.0102 |
|  | RP11-6N17.10 | 17 | rs1912155 | 393 | 2.5671 | 0.0103 |
|  | CFL1P5 | 5 | rs34221525 | 237 | -2.536 | 0.01121 |
|  | PNKP | 19 | rs2303758 | 326 | -2.52257 | 0.01165 |
|  | GGTA1P | 9 | rs177698 | 445 | -2.50565 | 0.01222 |
|  | RP11-254F7.2 | 2 | rs10495570 | 555 | -2.5043 | 0.01227 |
|  | TMTC1 | 12 | rs302371 | 760 | -2.5022 | 0.01234 |
|  | ARHGAP22 | 10 | rs12777174 | 606 | 2.4939 | 0.0126 |
|  | ARFGAP3 | 22 | rs2179258 | 532 | -2.4671 | 1.36E-02 |
|  | FAM189B | 1 | rs951241 | 305 | 2.46509 | 0.0137 |
|  | WNT11 | 11 | rs1944438 | 442 | -2.4571 | 0.014 |
|  | ZNF708 | 19 | rs1520068 | 279 | 2.4455 | 0.01447 |
|  | NEDD1 | 12 | rs7309474 | 579 | -2.4358 | 0.01486 |
|  | CD55 | 1 | rs4348726 | 373 | 2.4249 | 0.01531 |
|  | KLHL12 | 1 | rs6699298 | 471 | 2.4202 | 0.01551 |
|  | LSS | 21 | rs2839274 | 476 | -2.4178 | 0.0156 |
|  | SRR | 17 | rs1885987 | 406 | -2.413 | 0.0158 |
|  | RP11-656E20.5 | 12 | rs765790 | 445 | 2.4095 | 0.01598 |
|  | RP11-611E13.2 | 12 | rs710711 | 763 | 2.4076 | 0.01606 |
|  | RP11-448G15.3 | 4 | rs4235358 | 558 | 2.4008 | 0.01636 |
|  | TFIP11 | 22 | rs16982302 | 774 | 2.3888 | 1.69E-02 |
|  | NT5C3B | 17 | rs11653427 | 399 | -2.3818 | 0.0172 |
|  | DHRS4-AS1 | 14 | rs2277479 | 509 | 2.3729 | 0.01765 |
|  | CTD-3099C6.7 | 19 | rs34312893 | 428 | 2.37094 | 0.01774 |
|  | RP11-535A19.1 | 11 | rs1944438 | 436 | -2.363 | 0.0181 |
|  | DGAT2 | 11 | rs1944438 | 454 | -2.3561 | 0.0185 |
|  | MYO15B | 17 | rs820242 | 333 | -2.344 | 0.0191 |
|  | GIMAP4 | 7 | rs956642 | 421 | 2.3313 | 0.0197 |
|  | FPR3 | 19 | rs7256273 | 599 | 2.312 | 0.02078 |
|  | RP11-32B11.2 | 9 | rs10901089 | 518 | 2.3117 | 0.02079 |
|  | CTD-2636A23.2 | 5 | rs12655504 | 311 | 2.3116 | 0.0208 |
|  | LRRN1 | 3 | rs4349487 | 633 | 2.30815 | 0.02099 |
|  | MBLAC1 | 7 | rs3735453 | 265 | -2.305 | 0.0212 |
|  | SCPEP1 | 17 | rs792379 | 509 | 2.3032 | 0.0213 |
|  | AP001469.5 | 21 | rs2839274 | 464 | -2.3021 | 0.0213 |
|  | SENP7 | 3 | rs7626110 | 461 | 2.2991 | 0.0215 |
|  | KLHL11 | 17 | rs11653427 | 397 | -2.2888 | 0.0221 |
|  | SAMD10 | 20 | rs8114282 | 345 | -2.2829 | 0.0224 |
|  | SURF1 | 9 | rs736417 | 552 | 2.27913 | 0.02266 |
|  | RP11-459I19.1 | 2 | rs11695967 | 364 | -2.2677 | 0.02335 |
|  | PLA2G4C | 19 | rs10410302 | 502 | -2.26115 | 0.02375 |
|  | PRDM4 | 12 | rs7296362 | 464 | -2.2596 | 0.02385 |
|  | BNIP1 | 5 | rs3797456 | 566 | -2.2589 | 0.02389 |
|  | LRRC61 | 7 | rs11772167 | 344 | 2.2517 | 0.0243 |
|  | CCDC125 | 5 | rs34221525 | 250 | -2.2383 | 0.0252 |
|  | ERVK13-1 | 16 | rs12917910 | 290 | -2.23703 | 0.02528 |
|  | CHD1L | 1 | rs2352866 | 427 | 2.228 | 0.02588 |
|  | RP11-218M22.1 | 12 | rs8181744 | 452 | -2.2221 | 0.02627 |
|  | RP11-71H17.9 | 3 | rs652046 | 614 | -2.21473 | 0.02678 |
|  | ELP2 | 18 | rs1786224 | 509 | -2.213 | 0.0269 |
|  | RP11-867G23.12 | 11 | rs17582367 | 294 | 2.1888 | 0.0286 |
|  | GBA | 1 | rs951241 | 315 | -2.1781 | 0.0294 |
|  | SFMBT2 | 10 | rs7080582 | 865 | -2.17752 | 0.0294 |
|  | GHRLOS | 3 | rs17609118 | 590 | 2.17528 | 0.02961 |
|  | HLA-DQA1 | 6 | rs17429444 | 210 | -2.1745 | 0.02967 |
|  | IL18RAP | 2 | rs1997502 | 569 | 2.1703 | 0.02998 |
|  | RIN1 | 11 | rs17582367 | 294 | 2.1675 | 0.0302 |
|  | TACC3 | 4 | rs1250115 | 261 | 2.1667 | 0.03026 |
|  | PRMT6 | 1 | rs12025693 | 456 | 2.15477 | 0.03118 |
|  | MVK | 12 | rs2268400 | 425 | -2.1341 | 0.03283 |
|  | FHL3 | 1 | rs12745452 | 459 | 2.13261 | 0.03296 |
|  | PRMT5-AS1 | 14 | rs8019439 | 551 | 2.1214 | 0.03389 |
|  | PRMT5 | 14 | rs8019439 | 554 | 2.1214 | 0.03389 |
|  | ZNF205 | 16 | rs12917910 | 363 | -2.12014 | 0.03399 |
|  | CCDC7 | 10 | rs2990984 | 346 | -2.11549 | 0.0344 |
|  | POLR1B | 2 | rs4299347 | 431 | 2.1148 | 0.03445 |
|  | NINJ2 | 12 | rs8181744 | 475 | -2.1124 | 0.03465 |
|  | SETD3 | 14 | rs1257668 | 581 | -2.1064 | 0.03517 |
|  | CENPQ | 6 | rs9473583 | 349 | -2.1 | 0.03573 |
|  | RP11-37B2.1 | 8 | rs218885 | 415 | 2.0973 | 0.03597 |
|  | LINC00176 | 20 | rs4809401 | 315 | -2.0854 | 0.037 |
|  | BBX | 3 | rs9828598 | 497 | 2.07996 | 0.03753 |
|  | POLR2J3 | 7 | rs2734605 | 228 | -2.0773 | 0.0378 |
|  | CRIPAK | 4 | rs1680057 | 326 | -2.07017 | 0.03844 |
|  | SF3A3 | 1 | rs12745452 | 472 | 2.06751 | 0.03869 |
|  | WDR6 | 3 | rs3212 | 256 | 2.0657 | 0.03886 |
|  | CCDC71 | 3 | rs3212 | 256 | 2.0657 | 0.03886 |
|  | EIF4EBP2 | 10 | rs7094128 | 648 | 2.05892 | 0.0395 |
|  | CTD-2369P2.8 | 19 | rs1673130 | 364 | -2.0557 | 0.03981 |
|  | FRA10AC1 | 10 | rs732867 | 538 | 2.0535 | 0.04 |
|  | CHCHD2 | 7 | rs9986837 | 259 | -2.0466 | 0.0407 |
|  | AC005104.3 | 2 | rs41342147 | 353 | -2.0456 | 0.0408 |
|  | RAPGEFL1 | 17 | rs2269457 | 290 | -2.0455 | 0.0408 |
|  | BSCL2 | 11 | rs7949030 | 332 | -2.0446 | 0.0409 |
|  | USP22 | 17 | rs4985855 | 207 | -2.0426 | 0.0411 |
|  | ZNF74 | 22 | rs673017 | 301 | 2.0377 | 4.16E-02 |
|  | WIPI2 | 7 | rs3750014 | 357 | 2.0358 | 0.0418 |
|  | RAB11FIP5 | 2 | rs6725882 | 273 | 2.0323 | 0.04212 |
|  | RAB11B-AS1 | 19 | rs17160489 | 331 | 2.02921 | 0.04244 |
|  | ABCA7 | 19 | rs12985692 | 431 | 2.0255 | 0.04282 |
|  | PXDN | 2 | rs13015040 | 505 | -2.0216 | 0.04322 |
|  | TMEM80 | 11 | rs6421975 | 449 | 2.0211 | 0.0433 |
|  | RP3-402G11.25 | 22 | rs4838857 | 379 | 2.0188 | 4.35E-02 |
|  | ME2 | 18 | rs625566 | 469 | 2.016 | 0.0438 |
|  | NUDCD3 | 7 | rs10230538 | 371 | -2.0091 | 0.0445 |
|  | RAP1GAP | 1 | rs12030578 | 430 | -2.00911 | 0.04453 |
|  | AC093162.5 | 2 | rs17761664 | 424 | -2.0081 | 0.04463 |
|  | METTL21B | 12 | rs7978954 | 341 | -2.0076 | 0.04469 |
|  | ZNRD1-AS1 | 6 | rs385492 | 38 | 2.007 | 0.04475 |
|  | AC091729.9 | 7 | rs6942989 | 378 | 2.0062 | 0.0448 |
|  | STYXL1 | 7 | rs6965172 | 324 | 1.9995 | 0.0456 |
|  | GNLY | 2 | rs17618119 | 466 | 1.9935 | 0.0462 |
|  | UBE2H | 7 | rs968404 | 439 | 1.9799 | 0.0477 |
|  | B4GALNT3 | 12 | rs10774021 | 437 | -1.9777 | 0.04796 |
|  | XXbac-BPG299F13.17 | 6 | rs2523590 | 168 | -1.97763 | 0.04797 |
|  | CCDC13 | 3 | rs33518 | 481 | -1.97327 | 0.04846 |
|  | C20orf194 | 20 | rs2740210 | 492 | -1.9687 | 0.049 |
|  | ASRGL1 | 11 | rs7949030 | 418 | -1.9655 | 0.0494 |
