# Supplementary material for: Transcriptome-wide association study identifies new susceptibility genes and pathways for spondyloarthritis
Source: J Orthop Surg Res. 2023 Sep 4;18:659. doi: 10.1186/s13018-023-04029-4 (PMC10478464; doi:10.1186/s13018-023-04029-4)
Supplement: Supplementary file 4 — Additional file 4: Table S2 GO terms identified by Metascape enriched for SpA [file 13018_2023_4029_MOESM4_ESM.docx]

**Supplementary Table 2. GO terms identified by Metascape enriched for SpA**

| Category | Description | Number | LogP | Genes |
| --- | --- | --- | --- | --- |
| Canonical Pathways | PID IGF1 PATHWAY | M125 | -4.344668724 | HRAS\|PDPK1\|RAF1\|YWHAZ\|NCK2 |
| Canonical Pathways | PID MET PATHWAY | M48 | -3.08973217 | HRAS\|PDPK1\|RAF1\|NCK2\|RANBP9\|AKT1S1 |
| Canonical Pathways | PID MTOR 4PATHWAY | M121 | -2.565366057 | HRAS\|PDPK1\|RAF1\|YWHAZ\|AKT1S1 |
| Canonical Pathways | PID KIT PATHWAY | M231 | -2.23334125 | HRAS\|PDPK1\|RAF1\|SOCS1 |
| Canonical Pathways | SIG IL4RECEPTOR IN B LYPHOCYTES | M1718 | -2.218160409 | PDPK1\|RAF1\|SOCS1 |
| Canonical Pathways | PID INTEGRIN A9B1 PATHWAY | M118 | -2.313680901 | NOS2\|TGM2\|PAOX |
| Canonical Pathways | PID RAS PATHWAY | M269 | -2.089052462 | HRAS\|RIN1\|RASA4 |
| CORUM | TNF-alpha/NF-kappa B signaling complex 5 | CORUM:5233 | -2.313680901 | NFKBIB\|LRPPRC\|POLR1B |
| GO Biological Processes | mitochondrial DNA metabolic process | GO:0032042 | -4.952993951 | DNA2\|LONP1\|POLG2\|PRIMPOL |
| GO Biological Processes | mitochondrial genome maintenance | GO:0000002 | -3.829200191 | DNA2\|LONP1\|POLG2\|PRIMPOL |
| GO Biological Processes | mitochondrial DNA replication | GO:0006264 | -3.529242807 | DNA2\|POLG2\|PRIMPOL |
| GO Biological Processes | mitochondrion organization | GO:0007005 | -4.582872241 | BID\|CNP\|COX7A1\|DNA2\|SURF1\|ZNF205\|LONP1\|TOMM70\|YME1L1\|POLG2\|WIPI2\|CHCHD2\|DMAC2\|MTFR1L\|COA8\|PNPT1\|PRIMPOL\|UQCC3 |
| GO Biological Processes | generation of precursor metabolites and energy | GO:0006091 | -4.454304441 | ADH4\|BID\|COX7A1\|CYB5A\|GBA\|IDH3A\|ME2\|PKM\|PPP1R3A\|SURF1\|POLG2\|NNT\|DMGDH\|CYB5R4\|MTFR1L\|MRPS36\|UQCC3 |
| GO Biological Processes | cellular respiration | GO:0045333 | -3.651381707 | BID\|COX7A1\|GBA\|IDH3A\|SURF1\|POLG2\|NNT\|MTFR1L\|MRPS36\|UQCC3 |
| GO Biological Processes | electron transport chain | GO:0022900 | -3.400254632 | BID\|COX7A1\|CYB5A\|GBA\|ME2\|SURF1\|POLG2\|DMGDH\|UQCC3 |
| GO Biological Processes | energy derivation by oxidation of organic compounds | GO:0015980 | -3.198641119 | BID\|COX7A1\|GBA\|IDH3A\|PPP1R3A\|SURF1\|POLG2\|NNT\|MTFR1L\|MRPS36\|UQCC3 |
| GO Biological Processes | aerobic respiration | GO:0009060 | -2.896254325 | BID\|COX7A1\|IDH3A\|SURF1\|NNT\|MTFR1L\|MRPS36\|UQCC3 |
| GO Biological Processes | response to thyroid hormone | GO:0097066 | -4.344668724 | C2\|CTSH\|GBA\|LMO2\|TOMM70 |
| GO Biological Processes | amino-acid betaine metabolic process | GO:0006577 | -2.80726247 | ALDH7A1\|BHMT\|DMGDH |
| GO Biological Processes | amine catabolic process | GO:0009310 | -2.173376346 | ALDH7A1\|DMGDH\|PAOX |
| GO Biological Processes | cellular biogenic amine catabolic process | GO:0042402 | -2.173376346 | ALDH7A1\|DMGDH\|PAOX |
| GO Biological Processes | cellular modified amino acid catabolic process | GO:0042219 | -2.010942142 | BHMT\|GOT2\|DMGDH |
| GO Biological Processes | histone H4-R3 methylation | GO:0043985 | -4.050875089 | PRMT5\|PRDM4\|PRMT6 |
| GO Biological Processes | methylation | GO:0032259 | -3.413919146 | BHMT\|TYMS\|PRMT5\|PRDM4\|WDR6\|BHMT2\|EEF1AKMT3\|TFB1M\|COQ3\|PRMT6\|SETD3\|PCMTD1\|SMYD1 |
| GO Biological Processes | histone arginine methylation | GO:0034969 | -3.274973266 | PRMT5\|PRDM4\|PRMT6 |
| GO Biological Processes | peptidyl-arginine modification | GO:0018195 | -3.24133133 | PRMT5\|PRDM4\|PADI4\|PRMT6 |
| GO Biological Processes | protein methylation | GO:0006479 | -3.149574371 | BHMT\|PRMT5\|PRDM4\|EEF1AKMT3\|PRMT6\|SETD3\|PCMTD1\|SMYD1 |
| GO Biological Processes | protein alkylation | GO:0008213 | -3.149574371 | BHMT\|PRMT5\|PRDM4\|EEF1AKMT3\|PRMT6\|SETD3\|PCMTD1\|SMYD1 |
| GO Biological Processes | peptidyl-arginine methylation | GO:0018216 | -2.887114505 | PRMT5\|PRDM4\|PRMT6 |
| GO Biological Processes | macromolecule methylation | GO:0043414 | -2.465121468 | BHMT\|PRMT5\|PRDM4\|WDR6\|EEF1AKMT3\|TFB1M\|PRMT6\|SETD3\|PCMTD1\|SMYD1 |
| GO Biological Processes | antigen processing and presentation | GO:0019882 | -3.495831774 | CTSH\|GBA\|HLA-B\|HLA-DQA1\|HLA-DQB1\|HLA-DQB2\|ERAP1 |
| GO Biological Processes | MHC class II protein complex assembly | GO:0002399 | -2.887114505 | HLA-DQA1\|HLA-DQB1\|HLA-DQB2 |
| GO Biological Processes | peptide antigen assembly with MHC class II protein complex | GO:0002503 | -2.887114505 | HLA-DQA1\|HLA-DQB1\|HLA-DQB2 |
| GO Biological Processes | antigen processing and presentation of peptide antigen | GO:0048002 | -2.834731179 | HLA-B\|HLA-DQA1\|HLA-DQB1\|HLA-DQB2\|ERAP1 |
| GO Biological Processes | immunoglobulin production involved in immunoglobulin-mediated immune response | GO:0002381 | -2.73178588 | HLA-DQA1\|HLA-DQB1\|HLA-DQB2\|TRAF3IP2 |
| GO Biological Processes | MHC protein complex assembly | GO:0002396 | -2.596254252 | HLA-DQA1\|HLA-DQB1\|HLA-DQB2 |
| GO Biological Processes | peptide antigen assembly with MHC protein complex | GO:0002501 | -2.596254252 | HLA-DQA1\|HLA-DQB1\|HLA-DQB2 |
| GO Biological Processes | antigen processing and presentation of exogenous peptide antigen via MHC class II | GO:0019886 | -2.089052462 | HLA-DQA1\|HLA-DQB1\|HLA-DQB2 |
| GO Biological Processes | antigen processing and presentation of peptide antigen via MHC class II | GO:0002495 | -2.010942142 | HLA-DQA1\|HLA-DQB1\|HLA-DQB2 |
| GO Biological Processes | lipoprotein biosynthetic process | GO:0042158 | -3.583542636 | APOB\|PPM1B\|WIPI2\|ZDHHC8\|PGAP1\|PGAP3\|PIGU |
| GO Biological Processes | protein lipidation | GO:0006497 | -2.923043765 | PPM1B\|WIPI2\|ZDHHC8\|PGAP1\|PGAP3\|PIGU |
| GO Biological Processes | lipoprotein metabolic process | GO:0042157 | -2.794787685 | APOB\|PPM1B\|WIPI2\|ZDHHC8\|PGAP1\|PGAP3\|PIGU |
| GO Biological Processes | GPI anchor biosynthetic process | GO:0006506 | -2.049273081 | PGAP1\|PGAP3\|PIGU |
| GO Biological Processes | GPI anchor metabolic process | GO:0006505 | -2.010942142 | PGAP1\|PGAP3\|PIGU |
| GO Biological Processes | regulation of apoptotic cell clearance | GO:2000425 | -3.529242807 | C2\|TGM2\|ABCA7 |
| GO Biological Processes | biomineral tissue development | GO:0031214 | -3.524682447 | BGLAP\|CLEC3B\|WNT11\|ITGB1BP1\|TFIP11\|CNNM4\|FAM20A |
| GO Biological Processes | biomineralization | GO:0110148 | -3.524682447 | BGLAP\|CLEC3B\|WNT11\|ITGB1BP1\|TFIP11\|CNNM4\|FAM20A |
| GO Biological Processes | negative regulation of protein modification process | GO:0031400 | -3.410324219 | GBA\|PDPK1\|PKIB\|NCK2\|SOCS1\|ITGB1BP1\|CDYL\|BAG5\|PNKP\|GTPBP4\|STYXL1\|PRMT6\|DBNDD1\|LRRK1\|CDK5RAP3\|AKT1S1\|MASTL\|DTX3L |
| GO Biological Processes | negative regulation of catalytic activity | GO:0043086 | -2.138025176 | GBA\|HRAS\|PDE6G\|PDPK1\|PKIB\|RAF1\|SOCS1\|ITGB1BP1\|BAG5\|RASA4\|PLA2R1\|TFIP11\|GPSM1\|STYXL1\|DBNDD1\|CDK5RAP3\|AKT1S1\|MASTL\|PPP1R14A\|DTX3L |
| GO Biological Processes | negative regulation of phosphate metabolic process | GO:0045936 | -2.044548092 | GBA\|PDPK1\|PKIB\|NCK2\|SOCS1\|ITGB1BP1\|ANKLE2\|STYXL1\|DBNDD1\|LRRK1\|CDK5RAP3\|AKT1S1\|MASTL |
| GO Biological Processes | negative regulation of phosphorus metabolic process | GO:0010563 | -2.036859196 | GBA\|PDPK1\|PKIB\|NCK2\|SOCS1\|ITGB1BP1\|ANKLE2\|STYXL1\|DBNDD1\|LRRK1\|CDK5RAP3\|AKT1S1\|MASTL |
| GO Biological Processes | regulation of dephosphorylation | GO:0035303 | -2.012932521 | GBA\|ANKLE2\|SMG5\|STYXL1\|CDK5RAP3\|MASTL |
| GO Biological Processes | RNA biosynthetic process | GO:0032774 | -3.288388534 | CDK7\|NFKBIB\|MED27\|MED16\|PRMT5\|TCFL5\|PRDM4\|RPAP1\|TFB1M\|ELP2\|PHRF1\|POLR1B\|PRIMPOL\|POLR2J3\|GTF2H2C |
| GO Biological Processes | transcription, DNA-templated | GO:0006351 | -2.95764376 | CDK7\|NFKBIB\|MED27\|MED16\|PRMT5\|TCFL5\|PRDM4\|RPAP1\|TFB1M\|ELP2\|PHRF1\|POLR1B\|POLR2J3\|GTF2H2C |
| GO Biological Processes | nucleic acid-templated transcription | GO:0097659 | -2.936433016 | CDK7\|NFKBIB\|MED27\|MED16\|PRMT5\|TCFL5\|PRDM4\|RPAP1\|TFB1M\|ELP2\|PHRF1\|POLR1B\|POLR2J3\|GTF2H2C |
| GO Biological Processes | nucleobase-containing compound biosynthetic process | GO:0034654 | -2.921198796 | AK4\|AMD1\|CDK7\|NFKBIB\|TYMS\|SLC25A16\|MED27\|MED16\|PRMT5\|TCFL5\|PRDM4\|POLG2\|RPAP1\|TFB1M\|ELP2\|PHRF1\|POLR1B\|PRIMPOL\|POLR2J3\|GTF2H2C\|UQCC3 |
| GO Biological Processes | heterocycle biosynthetic process | GO:0018130 | -2.430466987 | AK4\|AMD1\|CDK7\|NFKBIB\|TYMS\|SLC25A16\|MED27\|MED16\|PRMT5\|TCFL5\|PRDM4\|POLG2\|RPAP1\|TFB1M\|ELP2\|PHRF1\|POLR1B\|PRIMPOL\|POLR2J3\|GTF2H2C\|UQCC3 |
| GO Biological Processes | aromatic compound biosynthetic process | GO:0019438 | -2.374829843 | AK4\|AMD1\|CDK7\|NFKBIB\|TYMS\|SLC25A16\|MED27\|MED16\|PRMT5\|TCFL5\|PRDM4\|POLG2\|RPAP1\|TFB1M\|ELP2\|PHRF1\|POLR1B\|PRIMPOL\|POLR2J3\|GTF2H2C\|UQCC3 |
| GO Biological Processes | transcription by RNA polymerase II | GO:0006366 | -2.34550481 | CDK7\|MED27\|MED16\|TCFL5\|PRDM4\|RPAP1\|ELP2\|PHRF1\|POLR2J3 |
| GO Biological Processes | regulation of GTPase activity | GO:0043087 | -3.202417088 | HRAS\|MMUT\|RAP1GAP\|RSU1\|TGM2\|WNT11\|EVI5\|ITGB1BP1\|RASA4\|GPSM1\|RAPGEFL1\|LARS1\|ARHGAP22\|CCDC125 |
| GO Biological Processes | positive regulation of GTPase activity | GO:0043547 | -2.803112923 | HRAS\|MMUT\|RAP1GAP\|RSU1\|TGM2\|WNT11\|EVI5\|RAPGEFL1\|LARS1\|ARHGAP22\|CCDC125 |
| GO Biological Processes | positive regulation of hydrolase activity | GO:0051345 | -2.499969149 | BID\|CTSH\|HRAS\|HTR2B\|MMUT\|PDPK1\|RAP1GAP\|RSU1\|TGM2\|WNT11\|EVI5\|AIM2\|RAPGEFL1\|LARS1\|ARHGAP22\|KHDC1\|CCDC125 |
| GO Biological Processes | regulation of telomere maintenance via telomerase | GO:0032210 | -3.07986576 | PKIB\|CCT3\|PNKP\|SMG5\|NAF1 |
| GO Biological Processes | positive regulation of telomere maintenance via telomerase | GO:0032212 | -2.914714279 | PKIB\|CCT3\|PNKP\|NAF1 |
| GO Biological Processes | regulation of telomere maintenance via telomere lengthening | GO:1904356 | -2.802488725 | PKIB\|CCT3\|PNKP\|SMG5\|NAF1 |
| GO Biological Processes | positive regulation of telomere maintenance via telomere lengthening | GO:1904358 | -2.775363247 | PKIB\|CCT3\|PNKP\|NAF1 |
| GO Biological Processes | positive regulation of DNA biosynthetic process | GO:2000573 | -2.458918355 | PKIB\|CCT3\|POLG2\|PNKP\|NAF1 |
| GO Biological Processes | regulation of telomere maintenance | GO:0032204 | -2.288758885 | PKIB\|CCT3\|PNKP\|SMG5\|NAF1 |
| GO Biological Processes | positive regulation of telomere maintenance | GO:0032206 | -2.23334125 | PKIB\|CCT3\|PNKP\|NAF1 |
| GO Biological Processes | regulation of DNA biosynthetic process | GO:2000278 | -2.19346915 | PKIB\|CCT3\|POLG2\|PNKP\|SMG5\|NAF1 |
| GO Biological Processes | positive regulation of defense response to virus by host | GO:0002230 | -3.068963794 | AIM2\|TOMM70\|TRAF3IP2\|DTX3L |
| GO Biological Processes | activation of innate immune response | GO:0002218 | -2.867618641 | AIM2\|TOMM70\|LILRA2\|TBK1\|ZCCHC3 |
| GO Biological Processes | regulation of response to biotic stimulus | GO:0002831 | -2.866028421 | CD55\|HLA-B\|PPM1B\|SOCS1\|AIM2\|TOMM70\|TRAF3IP2\|LILRA2\|TBK1\|ERAP1\|ZCCHC3\|DTX3L\|NLRC3 |
| GO Biological Processes | regulation of defense response to virus by host | GO:0050691 | -2.569870278 | AIM2\|TOMM70\|TRAF3IP2\|DTX3L |
| GO Biological Processes | regulation of defense response to virus | GO:0050688 | -2.433411987 | PPM1B\|AIM2\|TOMM70\|TRAF3IP2\|DTX3L |
| GO Biological Processes | regulation of innate immune response | GO:0045088 | -2.284385626 | HLA-B\|SOCS1\|AIM2\|TOMM70\|LILRA2\|TBK1\|ERAP1\|ZCCHC3\|NLRC3 |
| GO Biological Processes | activation of immune response | GO:0002253 | -2.20814647 | C2\|CD55\|FPR3\|HLA-DQB1\|HRAS\|MASP1\|AIM2\|TOMM70\|LILRA2\|TBK1\|ZCCHC3 |
| GO Biological Processes | regulation of actin filament depolymerization | GO:0030834 | -3.042579554 | CAPG\|WDR1\|DSTN\|SPTBN5\|LIMA1 |
| GO Biological Processes | regulation of anatomical structure size | GO:0090066 | -2.4431479 | CAPG\|HTR2B\|NCK2\|ITGB1BP1\|WDR1\|DSTN\|KCNMB4\|SPTBN5\|TRPV2\|LIMA1\|LARS1\|SEMA5B\|SCPEP1\|SEMA4A\|AKT1S1 |
| GO Biological Processes | regulation of protein depolymerization | GO:1901879 | -2.115532547 | CAPG\|WDR1\|DSTN\|SPTBN5\|LIMA1 |
| GO Biological Processes | regulation of protein-containing complex disassembly | GO:0043244 | -2.092394301 | CAPG\|GBA\|WDR1\|DSTN\|SPTBN5\|LIMA1 |
| GO Biological Processes | proteolysis involved in cellular protein catabolic process | GO:0051603 | -3.022848977 | CTSH\|GBA\|PSMA7\|SGTA\|UBE2H\|USP1\|TRIM25\|LONP1\|MAEA\|YME1L1\|USP22\|FBXL4\|ANAPC4\|DMAC2\|HACE1\|MVB12B\|ZFAND2A\|CACUL1\|DTX3L |
| GO Biological Processes | cellular protein catabolic process | GO:0044257 | -2.782836138 | CTSH\|GBA\|PSMA7\|SGTA\|UBE2H\|USP1\|TRIM25\|LONP1\|MAEA\|YME1L1\|USP22\|FBXL4\|ANAPC4\|DMAC2\|HACE1\|MVB12B\|ZFAND2A\|CACUL1\|DTX3L |
| GO Biological Processes | protein catabolic process | GO:0030163 | -2.740618319 | APOB\|CTSH\|GBA\|PSMA7\|SGTA\|UBE2H\|USP1\|TRIM25\|LONP1\|MAEA\|YME1L1\|USP22\|FBXL4\|ANAPC4\|DMAC2\|HACE1\|MVB12B\|ZFAND2A\|CACUL1\|DTX3L |
| GO Biological Processes | modification-dependent macromolecule catabolic process | GO:0043632 | -2.737595663 | GBA\|SGTA\|UBE2H\|USP1\|TRIM25\|LONP1\|MAEA\|USP22\|FBXL4\|ANAPC4\|DMAC2\|HACE1\|PNPT1\|MVB12B\|ZFAND2A\|CACUL1\|DTX3L |
| GO Biological Processes | modification-dependent protein catabolic process | GO:0019941 | -2.43852192 | GBA\|SGTA\|UBE2H\|USP1\|TRIM25\|LONP1\|MAEA\|USP22\|FBXL4\|ANAPC4\|DMAC2\|HACE1\|MVB12B\|ZFAND2A\|CACUL1\|DTX3L |
| GO Biological Processes | ubiquitin-dependent protein catabolic process | GO:0006511 | -2.143410408 | GBA\|SGTA\|UBE2H\|USP1\|TRIM25\|MAEA\|USP22\|FBXL4\|ANAPC4\|DMAC2\|HACE1\|MVB12B\|ZFAND2A\|CACUL1\|DTX3L |
| GO Biological Processes | proteasomal protein catabolic process | GO:0010498 | -2.042070084 | GBA\|PSMA7\|SGTA\|UBE2H\|TRIM25\|MAEA\|FBXL4\|ANAPC4\|DMAC2\|HACE1\|ZFAND2A |
| GO Biological Processes | epithelial cell maturation | GO:0002070 | -2.972819158 | HOXA5\|TYMS\|TMEM79 |
| GO Biological Processes | carbohydrate derivative biosynthetic process | GO:1901137 | -2.864735283 | AK4\|AMD1\|EXTL1\|GALNT3\|GGTA1\|ST3GAL3\|TYMS\|SLC25A16\|B4GALT2\|ABCC5\|MAN1A2\|PGAP1\|TMTC1\|B3GNT9\|PGAP3\|PIGU\|UQCC3 |
| GO Biological Processes | carbohydrate metabolic process | GO:0005975 | -2.657197438 | GALNT3\|GGTA1\|IDH3A\|PDK1\|PKM\|PPP1R3A\|ST3GAL3\|B4GALT2\|MAN1A2\|COQ3\|ENOSF1\|DGAT2\|B3GNT9\|G6PC3\|GUSBP3 |
| GO Biological Processes | glycosylation | GO:0070085 | -2.236844505 | EXTL1\|GALNT3\|GBA\|GGTA1\|ST3GAL3\|B4GALT2\|MAN1A2\|TMTC1\|B3GNT9 |
| GO Biological Processes | cellular amino acid metabolic process | GO:0006520 | -2.753244365 | BHMT\|BPHL\|GOT2\|MMUT\|NOS2\|HIBADH\|BHMT2\|LARS1\|ENOSF1\|SRR\|ASRGL1 |
| GO Biological Processes | aspartate family amino acid biosynthetic process | GO:0009067 | -2.533763733 | BHMT\|GOT2\|BHMT2 |
| GO Biological Processes | aspartate family amino acid metabolic process | GO:0009066 | -2.325696655 | BHMT\|GOT2\|BHMT2\|ASRGL1 |
| GO Biological Processes | alpha-amino acid metabolic process | GO:1901605 | -2.215721389 | BHMT\|GOT2\|MMUT\|NOS2\|HIBADH\|BHMT2\|SRR\|ASRGL1 |
| GO Biological Processes | sulfur amino acid metabolic process | GO:0000096 | -2.010942142 | BHMT\|MMUT\|BHMT2 |
| GO Biological Processes | adaptive immune response based on somatic recombination of immune receptors built from immunoglobulin superfamily domains | GO:0002460 | -2.743931063 | C2\|CTSH\|CD55\|HLA-DQA1\|HLA-DQB1\|HLA-DQB2\|HRAS\|IL18R1\|TRAF3IP2\|SEMA4A |
| GO Biological Processes | T-helper 1 type immune response | GO:0042088 | -2.596254252 | HRAS\|IL18R1\|SEMA4A |
| GO Biological Processes | protein targeting | GO:0006605 | -2.65133062 | BID\|SCARB2\|SGTA\|SRP19\|YWHAZ\|TOMM70\|NACAD\|ZNF205-AS1\|ZFAND2A\|RAB3IP |
| GO Biological Processes | protein targeting to ER | GO:0045047 | -2.459785511 | SGTA\|SRP19\|ZNF205-AS1\|ZFAND2A |
| GO Biological Processes | protein localization to endoplasmic reticulum | GO:0070972 | -2.408323217 | SGTA\|SRP19\|KDELR2\|ZNF205-AS1\|ZFAND2A |
| GO Biological Processes | establishment of protein localization to endoplasmic reticulum | GO:0072599 | -2.325696655 | SGTA\|SRP19\|ZNF205-AS1\|ZFAND2A |
| GO Biological Processes | establishment of protein localization to membrane | GO:0090150 | -2.018817001 | BID\|SGTA\|SRP19\|TOMM70\|NACAD\|EMC9\|ZNF205-AS1\|RAB3IP |
| GO Biological Processes | lipid storage | GO:0019915 | -2.648479571 | GBA\|PLA2G4C\|BSCL2\|DGAT2 |
| GO Biological Processes | lipid localization | GO:0010876 | -2.416368865 | APOB\|SCARB2\|GBA\|GOT2\|NOS2\|PLA2G4C\|ABCC3\|ABCA7\|ABCA6\|BSCL2\|LIMA1\|DGAT2 |
| GO Biological Processes | regulation of generation of precursor metabolites and energy | GO:0043467 | -2.563775975 | AK4\|COX7A1\|NOS2\|PPP1R3A\|CHCHD2\|COA8\|PNPT1 |
| GO Biological Processes | regulation of cellular respiration | GO:0043457 | -2.29418391 | AK4\|COX7A1\|NOS2\|PNPT1 |
| GO Biological Processes | tricarboxylic acid cycle | GO:0006099 | -2.049273081 | IDH3A\|NNT\|MRPS36 |
| GO Biological Processes | regulation of cellular localization | GO:0060341 | -2.44299226 | BTF3\|HRAS\|PCNT\|PDPK1\|CCT3\|REEP5\|STX7\|ITGB1BP1\|ABCA7\|MCRS1\|MESD\|RAB11FIP5\|ZDHHC8\|CENPQ\|JPH2\|SELENON\|PGAP1\|CDK5RAP3\|PARD6G\|REEP6\|DTX3L |
| GO Biological Processes | mRNA 3'-end processing | GO:0031124 | -2.424985787 | ZC3H3\|RPRD1B\|PNPT1\|MBLAC1 |
| GO Biological Processes | plasma lipoprotein particle assembly | GO:0034377 | -2.418299854 | APOB\|ABCA7\|ZDHHC8 |
| GO Biological Processes | protein-lipid complex assembly | GO:0065005 | -2.264875762 | APOB\|ABCA7\|ZDHHC8 |
| GO Biological Processes | regulation of plasma lipoprotein particle levels | GO:0097006 | -2.147115017 | APOB\|ABCA7\|ZDHHC8\|DGAT2 |
| GO Biological Processes | cell division | GO:0051301 | -2.402490434 | CDK7\|SEPTIN2\|AURKC\|EVI5\|MAEA\|TACC3\|KATNA1\|ANKLE2\|ANAPC4\|CABLES2\|PARD6G\|MASTL\|NEDD1\|SGO2\|NUP43 |
| GO Biological Processes | regulation of mitochondrial translation | GO:0070129 | -2.364754661 | TSFM\|LRPPRC\|MTG1 |
| GO Biological Processes | regulation of mitochondrial gene expression | GO:0062125 | -2.173376346 | TSFM\|LRPPRC\|MTG1 |
| GO Biological Processes | aging | GO:0007568 | -2.360743187 | BGLAP\|CNP\|GBA\|HRAS\|IGFBP2\|PITX3\|TYMS\|PLA2R1\|PRMT6\|SRR |
| GO Biological Processes | glutamine family amino acid catabolic process | GO:0009065 | -2.313680901 | GOT2\|NOS2\|ASRGL1 |
| GO Biological Processes | cellular nitrogen compound catabolic process | GO:0044270 | -2.296636158 | BHMT\|CNP\|DNA2\|PDE4C\|SMG5\|SND1\|DMGDH\|NUDT9\|EXOSC5\|PNPT1\|NT5C3B |
| GO Biological Processes | retrograde vesicle-mediated transport, Golgi to endoplasmic reticulum | GO:0006890 | -2.29418391 | BNIP1\|KDELR2\|DNAJC28\|RAB6C |
| GO Biological Processes | cellular response to DNA damage stimulus | GO:0006974 | -2.285792424 | BID\|CDK7\|DNA2\|MCM4\|RAD17\|TNP1\|USP1\|CHD1L\|MCRS1\|POLG2\|PNKP\|TFIP11\|PRMT6\|CDK5RAP3\|MASTL\|DTX3L\|CCDC13\|PRIMPOL\|GTF2H2C |
| GO Biological Processes | DNA repair | GO:0006281 | -2.199678678 | CDK7\|DNA2\|MCM4\|RAD17\|TNP1\|USP1\|CHD1L\|MCRS1\|POLG2\|PNKP\|PRMT6\|DTX3L\|PRIMPOL\|GTF2H2C |
| GO Biological Processes | DNA metabolic process | GO:0006259 | -2.001503554 | CDK7\|DNA2\|MCM4\|RAD17\|TNP1\|TYMS\|USP1\|LONP1\|CHD1L\|MCRS1\|POLG2\|PNKP\|TFIP11\|PRMT6\|EXOSC5\|DTX3L\|PRIMPOL\|GTF2H2C |
| GO Biological Processes | positive regulation of protein processing | GO:0010954 | -2.264875762 | MELTF\|CLEC3B\|TNP1 |
| GO Biological Processes | positive regulation of protein maturation | GO:1903319 | -2.173376346 | MELTF\|CLEC3B\|TNP1 |
| GO Biological Processes | isoprenoid metabolic process | GO:0006720 | -2.264624545 | ADH4\|LSS\|MVK\|RARRES2\|SCPEP1\|DGAT2 |
| GO Biological Processes | secondary alcohol metabolic process | GO:1902652 | -2.044222472 | APOB\|GBA\|IDH3A\|LSS\|MVK\|LIMA1 |
| GO Biological Processes | mitochondrial gene expression | GO:0140053 | -2.243489503 | TSFM\|TFB1M\|FASTKD5\|MRPS14\|PNPT1 |
| GO Biological Processes | regulation of RNA stability | GO:0043487 | -2.061418088 | RNASEL\|TRAF3IP2\|EXOSC5\|FASTKD5\|PNPT1\|NAF1\|NT5C3B |
| GO Biological Processes | lens fiber cell differentiation | GO:0070306 | -2.173376346 | PITX3\|PLAAT1\|WNT5B |
| GO Biological Processes | maturation of LSU-rRNA | GO:0000470 | -2.173376346 | NSA2\|GTPBP4\|WDR12 |
| GO Biological Processes | liver regeneration | GO:0097421 | -2.130382437 | TYMS\|PRMT5\|PNPT1 |
| GO Biological Processes | endoplasmic reticulum organization | GO:0007029 | -2.15693961 | BNIP1\|SGTA\|REEP5\|EMC9\|REEP6 |
| GO Biological Processes | divalent inorganic cation homeostasis | GO:0072507 | -2.13052522 | CD55\|FPR3\|HTR2B\|PDPK1\|TGM2\|CNNM4\|CNNM3\|TRPV2\|FAM20A\|RMDN3\|CCL28\|JPH2\|SELENON\|TMEM178A |
| GO Biological Processes | regulation of double-strand break repair via nonhomologous end joining | GO:2001032 | -2.130382437 | PNKP\|TFIP11\|DTX3L |
| GO Biological Processes | regulation of interferon-beta production | GO:0032648 | -2.092699281 | PPM1B\|TOMM70\|TBK1\|NLRC3 |
| GO Biological Processes | regulation of I-kappaB kinase/NF-kappaB signaling | GO:0043122 | -2.026186065 | HTR2B\|NFKBIB\|PDPK1\|PPM1B\|TRIM25\|TRAF3IP2\|TBK1\|TMEM101\|NLRC3 |
| GO Biological Processes | mitotic spindle organization | GO:0007052 | -2.055595615 | PCNT\|AURKC\|TACC3\|TUBG2\|TOGARAM2 |
| GO Biological Processes | positive regulation of T cell mediated immunity | GO:0002711 | -2.040534043 | CD55\|HLA-B\|STX7\|IL18R1 |
| GO Biological Processes | negative regulation of intracellular signal transduction | GO:1902532 | -2.02441186 | BID\|FHL2\|GBA\|PPM1B\|ITGB1BP1\|BAG5\|RANBP9\|RASA4\|ABCA7\|DUSP13\|CDK5RAP3\|AKT1S1\|NLRC3\|CCDC125\|CASTOR1 |
| GO Biological Processes | protein localization to cell surface | GO:0034394 | -2.010942142 | WNT11\|MESD\|PIGU |
| KEGG Pathway | Axon guidance | hsa04360 | -3.612745897 | HRAS\|PDPK1\|RAF1\|NCK2\|SEMA5B\|SEMA4A\|ROBO3\|WNT5B\|PARD6G\|NTNG2 |
| KEGG Pathway | mTOR signaling pathway | hsa04150 | -2.823950553 | HRAS\|PDPK1\|RAF1\|WNT11\|ATP6V1H\|WNT5B\|AKT1S1\|CASTOR1 |
| KEGG Pathway | Insulin signaling pathway | hsa04910 | -2.510182943 | HRAS\|PDPK1\|PPP1R3A\|PRKAB2\|RAF1\|SOCS1\|G6PC3 |
| KEGG Pathway | Toxoplasmosis | hsa05145 | -2.320127083 | HLA-DQA1\|HLA-DQB1\|NFKBIB\|NOS2\|PDPK1\|SOCS1 |
| KEGG Pathway | Glycine, serine and threonine metabolism | hsa00260 | -2.648479571 | ALDH7A1\|BHMT\|DMGDH\|SRR |
| KEGG Pathway | Viral myocarditis | hsa05416 | -3.73837646 | BID\|CD55\|HLA-B\|HLA-DQA1\|HLA-DQB1\|SGCB |
| KEGG Pathway | Pyruvate metabolism | hsa00620 | -2.391062863 | ADH4\|ALDH7A1\|ME2\|PKM |
| KEGG Pathway | Various types of N-glycan biosynthesis | hsa00513 | -2.689514338 | ST3GAL3\|B4GALT2\|MAN1A2\|B4GALNT3 |
| KEGG Pathway | Cysteine and methionine metabolism | hsa00270 | -2.29418391 | AMD1\|BHMT\|GOT2\|BHMT2 |
| KEGG Pathway | Staphylococcus aureus infection | hsa05150 | -2.651934098 | C2\|FPR3\|HLA-DQA1\|HLA-DQB1\|KRT32\|MASP1 |
| KEGG Pathway | Influenza A | hsa05164 | -2.557639254 | BID\|HLA-DQA1\|HLA-DQB1\|NFKBIB\|RAF1\|RNASEL\|TRIM25\|TBK1 |
| KEGG Pathway | Herpes simplex virus 1 infection | hsa05168 | -2.378424349 | BID\|HLA-B\|HLA-DQA1\|HLA-DQB1\|RNASEL\|ZNF10\|ZNF708\|ZNF43\|ZNF74\|ZNF205\|TBK1\|ZNF611\|ZNF100\|ZNF713\|ZNF429 |
| KEGG Pathway | ABC transporters | hsa02010 | -2.459785511 | ABCC3\|ABCC5\|ABCA7\|ABCA6 |
| KEGG Pathway | Antifolate resistance | hsa01523 | -2.049273081 | TYMS\|ABCC3\|ABCC5 |
| KEGG Pathway | Arginine and proline metabolism | hsa00330 | -2.263408658 | AMD1\|ALDH7A1\|GOT2\|NOS2 |
| Reactome Gene Sets | Choline catabolism | R-HSA-6798163 | -4.289413374 | ALDH7A1\|BHMT\|DMGDH |
| Reactome Gene Sets | Interferon gamma signaling | R-HSA-877300 | -2.770142871 | HLA-B\|HLA-DQA1\|HLA-DQB1\|HLA-DQB2\|TRIM25\|SOCS1 |
| Reactome Gene Sets | Interferon Signaling | R-HSA-913531 | -2.711955518 | HLA-B\|HLA-DQA1\|HLA-DQB1\|HLA-DQB2\|PPM1B\|RNASEL\|TRIM25\|SOCS1\|NUP43 |
| Reactome Gene Sets | Translocation of ZAP-70 to Immunological synapse | R-HSA-202430 | -2.662375899 | HLA-DQA1\|HLA-DQB1\|HLA-DQB2 |
| Reactome Gene Sets | Phosphorylation of CD3 and TCR zeta chains | R-HSA-202427 | -2.47454776 | HLA-DQA1\|HLA-DQB1\|HLA-DQB2 |
| Reactome Gene Sets | PD-1 signaling | R-HSA-389948 | -2.418299854 | HLA-DQA1\|HLA-DQB1\|HLA-DQB2 |
| Reactome Gene Sets | Adaptive Immune System | R-HSA-1280218 | -3.713535149 | CTSH\|HLA-B\|HLA-DQA1\|HLA-DQB1\|HLA-DQB2\|HRAS\|KLC1\|LMO7\|NFKBIB\|PDPK1\|PSMA7\|RAF1\|RAP1GAP\|UBE2H\|YWHAZ\|SOCS1\|LILRA2\|FBXL4\|ANAPC4\|FBXO40\|ERAP1\|KLHL11\|HACE1\|DTX3L |
| Reactome Gene Sets | Class I MHC mediated antigen processing & presentation | R-HSA-983169 | -2.166112365 | HLA-B\|LMO7\|PSMA7\|UBE2H\|SOCS1\|FBXL4\|ANAPC4\|FBXO40\|ERAP1\|KLHL11\|HACE1\|DTX3L |
| Reactome Gene Sets | Post-translational modification: synthesis of GPI-anchored proteins | R-HSA-163125 | -2.017010876 | MELTF\|PGAP1\|NTNG2\|PIGU\|SPRN |
| Reactome Gene Sets | Rap1 signalling | R-HSA-392517 | -2.887114505 | RAF1\|RAP1GAP\|YWHAZ |
| Reactome Gene Sets | Acyl chain remodelling of PI | R-HSA-1482922 | -2.80726247 | PLA2G4C\|PLA2R1\|MBOAT7 |
| Reactome Gene Sets | Acyl chain remodelling of PE | R-HSA-1482839 | -2.089052462 | PLA2G4C\|PLA2R1\|PLAAT1 |
| Reactome Gene Sets | Metabolism of amino acids and derivatives | R-HSA-71291 | -2.624585513 | AMD1\|ALDH7A1\|BHMT\|GOT2\|PSMA7\|HIBADH\|BHMT2\|DMGDH\|RPL26L1\|LARS1\|SRR\|ASRGL1\|PAOX |
| Reactome Gene Sets | Sulfur amino acid metabolism | R-HSA-1614635 | -2.173376346 | BHMT\|GOT2\|BHMT2 |
| Reactome Gene Sets | Citric acid cycle (TCA cycle) | R-HSA-71403 | -2.47454776 | IDH3A\|ME2\|NNT |
| Reactome Gene Sets | Pyruvate metabolism and Citric Acid (TCA) cycle | R-HSA-71406 | -2.147115017 | IDH3A\|ME2\|PDK1\|NNT |
| Reactome Gene Sets | Interconversion of nucleotide di- and triphosphates | R-HSA-499943 | -2.089052462 | AK4\|TYMS\|NME1-NME2 |
| Reactome Gene Sets | Glucose metabolism | R-HSA-70326 | -2.055595615 | GOT2\|PKM\|SLC37A1\|G6PC3\|NUP43 |
| WikiPathways | Prolactin signaling pathway | WP2037 | -3.178933663 | HRAS\|NFKBIB\|NOS2\|RAF1\|YWHAZ\|SOCS1 |
| WikiPathways | Serotonin receptor 2 and ELK-SRF/GATA4 signaling | WP732 | -2.596254252 | HRAS\|HTR2B\|RAF1 |
| WikiPathways | Interleukin-11 signaling pathway | WP2332 | -2.495503018 | BGLAP\|HRAS\|PDPK1\|RAF1 |
| WikiPathways | T-cell activation SARS-CoV-2 | WP5098 | -2.136086603 | HRAS\|PDPK1\|RAF1\|IL18R1\|CCL28 |
| WikiPathways | Nitric oxide metabolism in cystic fibrosis | WP4947 | -3.165511222 | NOS2\|PRMT5\|PRMT6 |
| WikiPathways | Allograft Rejection | WP2328 | -3.613570063 | C2\|CD55\|HLA-B\|HLA-DQA1\|HLA-DQB1\|GNLY\|BHMT2 |
| WikiPathways | Amino acid metabolism | WP3925 | -3.553916467 | ADH4\|ALDH7A1\|BHMT\|GOT2\|MMUT\|PKM\|HIBADH |
| WikiPathways | EPO receptor signaling | WP581 | -2.264875762 | PDK1\|RAF1\|SOCS1 |
| WikiPathways | Fragile X syndrome | WP4549 | -2.125365444 | EIF4EBP2\|PDK1\|RAF1\|RAP1GAP\|HOMER1\|AKT1S1 |
| WikiPathways | Photodynamic therapy-induced HIF-1 survival signaling | WP3614 | -2.775363247 | BID\|IGFBP2\|NOS2\|PKM |
| WikiPathways | One-carbon metabolism and related pathways | WP3940 | -2.175219972 | BHMT\|TYMS\|BHMT2\|DMGDH |
| WikiPathways | Complement activation | WP545 | -2.47454776 | C2\|CD55\|MASP1 |
| WikiPathways | 1q21.1 copy number variation syndrome | WP4905 | -2.173376346 | FMO5\|PRKAB2\|CHD1L |
| WikiPathways | Autophagy | WP4923 | -2.089052462 | PRKAB2\|WIPI2\|AKT1S1 |
| WikiPathways | Pyrimidine metabolism | WP4022 | -2.15693961 | TYMS\|POLR1B\|PNPT1\|POLR2J3\|NME1-NME2 |
